# An integrated system for identifying the hidden assassins in traditional medicines containing aristolochic acids

**DOI:** 10.1038/srep11318

**Published:** 2015-08-13

**Authors:** Lan Wu, Wei Sun, Bo Wang, Haiyu Zhao, Yaoli Li, Shaoqing Cai, Li Xiang, Yingjie Zhu, Hui Yao, Jingyuan Song, Yung-Chi Cheng, Shilin Chen

**Affiliations:** 1 Institute of Chinese Materia Medica, China Academy of Chinese Medical Sciences, Beijing, China; 2 College of Pharmacy, Hubei University of Chinese Medicine, Wuhan, China; 3 Institute of Medicinal Plant Development, Chinese Academy of Medical Sciences & Peking Union Medical College, Beijing, China; 4 State Key Laboratory of Natural and Biomimetic Drugs, School of Pharmaceutical Sciences, Peking University Health Science Centre, Beijing, China; 5 Department of Pharmacology, Yale University School of Medicine, New Haven, CT 06510, USA

## Abstract

Traditional herbal medicines adulterated and contaminated with plant materials from the Aristolochiaceae family, which contain aristolochic acids (AAs), cause aristolochic acid nephropathy. Approximately 256 traditional Chinese patent medicines, containing Aristolochiaceous materials, are still being sold in Chinese markets today. In order to protect consumers from health risks due to AAs, the hidden assassins, efficient methods to differentiate Aristolochiaceous herbs from their putative substitutes need to be established. In this study, 158 Aristolochiaceous samples representing 46 species and four genera as well as 131 non-Aristolochiaceous samples representing 33 species, 20 genera and 12 families were analyzed using DNA barcodes based on the ITS2 and *psbA-trnH* sequences. Aristolochiaceous materials and their non-Aristolochiaceous substitutes were successfully identified using BLAST1, the nearest distance method and the neighbor-joining (NJ) tree. In addition, based on sequence information of ITS2, we developed a Real-Time PCR assay which successfully identified herbal material from the Aristolochiaceae family. Using Ultra High Performance Liquid Chromatography-Mass Spectrometer (UHPLC-HR-MS), we demonstrated that most representatives from the Aristolochiaceae family contain toxic AAs. Therefore, integrated DNA barcodes, Real-Time PCR assays using TaqMan probes and UHPLC-HR-MS system provides an efficient and reliable authentication system to protect consumers from health risks due to the hidden assassins (AAs).

Traditional herbal medicine is recognized as an important component of primary healthcare by the WHO. However, many people remain unaware of the risks related to some traditional herbal prescription medicines that could contain aristolochic acids (AAs), often referred to as “the hidden assassins”. Aristolochic acids (AAs), found in plants from the Aristolochiaceae family, belong to a nephrotoxic and carcinogenic group of chemicals that cause significant upper tract urothelial carcinomas (UTUC). AAs have the ability to react with DNA to form covalent dA-aristolactam (AL) and dG-AL adducts^1^,^2^. Current evidence from AA-induced mutagenesis studies revealed that the mutation rates caused by AAs were higher than the rate of mutation attributed to other carcinogens such as tobacco and ultraviolet light^3^. Traditional herbal medicines adulterated and contaminated with plant materials from the Aristolochiaceae family, which contain AAs, have caused a number of large epidemic events. In the early 1990s, an epidemic of urothelial carcinomas related to AAs occurred in Belgium, where a number of women were prescribed the Chinese herb Fangji (*Stephania tetrandra* S. Moore) for weight-loss, which was incorrectly substituted with AAs containing Guangfangji (*Aristolochia fangchi* Y. C. Wu ex L. D. Chow & S. M. Hwang). Subsequently, the women developed renal interstitial fibrosis and upper tract urothelial carcinomas-aristolochic acid nephropathy (UTUC-AAN)^4^.

In another case, patients prescribed herbal preparations for the treatment of eczema suffered from interstitial nephritis, when preparations contained AAs. The main reason for this was the incorrect substitution of adulterants such as Guanmutong (*Ar. manshuriensis* Kom.) instead of Mutong (*Akebia quinata* (Houtt.) Decne.) and Guangfangji (*Ar. fangchi*) instead of Fangji (*Stephania tetrandra*)^5^.

Balkan endemic nephropathy (BEN) is a chronic tubulointerstitial disease associated with urothelial atypia and is observed in patients commonly residing in parts of southeastern Europe^6^. Patients with AAN and BEN present with very similar symptoms, and there is a growing body of evidence that suggests that both diseases are caused by AAs^7^. For BEN, the exposure of patients to AA containing compounds has been shown to occur through the contamination of seeds from *Aristolochia* plants grown in local wheat fields, commingling with the harvested wheat grain, and consequently contaminating wheat flour and bread^8^. Therefore, health risks could potentially occur when herbs containing AAs contaminate grain and other herbal products with no mention or knowledge of Aristolochiaceous material being present^6^.

There has also been a report of chronic interstitial fibrosis related to the ingestion of Indian herbal medicines that contain AAs^9^. So far, AAN has been reported in many countries but its true incidence is unknown and what is currently known is probably only the tip of the iceberg^10^,^11^,^12^.

Since 2001, AAs-containing herbal preparations were banned in Europe, North America, Taiwan, and Hong Kong. Subsequently, China Food and Drug Administration (CFDA) has prohibited the use of Chinese medicinal materials of Qingmuxiang (the roots and rhizomes of *Ar. debilis* and *Ar. contorta*), Guangfangji (the root and rhizome of *Ar. fangchi*) and Guanmutong (the stem of *Ar. manshuriensis*) in herbal materials and traditional Chinese patent medicines (http://www.sda.gov.cn/WS01/CL0844/9977.html; http://www.sda.gov.cn/WS01/CL0844/10242.html). The CFDA issued that traditional Chinese patent medicines containing Madouling (the fruits of *Ar. debilis* and *Ar. contorta*), Tianxianteng (the herbs of *Ar. debilis* and *Ar. contorta*), Xungufeng (the herbs of *Ar. mollissima*), and Zhushalian (the root and rhizome of *Ar. tuberosa*) should be under surveillance as prescription drugs (http://www.sda.gov.cn/WS01/CL0844/10242.html). Furthermore, substitution of AAs-containing herbs with AAs free herbs in Chinese herbal products is highly recommended (http://www.sda.gov.cn/WS01/CL0844/10242.html). However, the Pharmacopoeia of the People’s Republic of China 2010 never banned the use of three types of herbs, Madouling (the fruits of *Ar. debilis* and *Ar. contorta*), Tianxianteng (the herbs of *Ar. debilis* and *Ar. contorta*), and Xixin (the roots and rhizomes of *Asarum heterotropoides* var. *mandshuricum*, *As. sieboldii*, and *As. sieboldii* var. *seoulense*)^13^. Approximately 256 traditional Chinese patent medicines containing Aristolochiaceous materials are still sold in the Chinese market today (Data from *the Drug Standard of Ministry of Health People’s Republic of China, Chinese Pharmacopoeia and Drug Standard of China Food and Drug Administration*, etc.) (Table 1; additional details in Supplementary Table S1 online).

It has been reported that the main confusion with Aristolochiaceous materials adulterated into herbal medicines is due to the similar Chinese vernacular and morphology. However, traditional method of identification of medicinal plants mostly depends on the macroscopic and microscopic characteristics, yet, dry herbs and raw materials cannot be precisely determined based on morphological characteristics. As a result, contamination and possible herbal adulteration or substitution exists in the marketplace, and is a possible threat to the consumer. In recent years, DNA barcoding technology has been widely applied in species identification and wildlife forensic identification due to the advantage of strong universality and good repeatability^14^,^15^,^16^,^17^,^18^,^19^,^20^,^21^,^22^,^23^. Based on systematic research, researchers have proposed the internal transcribed spacer 2 (ITS2) as the core and *psbA-trnH* as a supplementary DNA barcode, for medicinal plant authentication^24^,^25^. Discriminatory capabilities of ITS2 and *psbA-trnH* sequences have been validated by many previous studies^26^,^27^,^28^,^29^,^30^,^31^. For identification of Aristolochiaceous herbs and their non-Aristolochiaceous substitutes, previous barcoding using the nr-DNA ITS gene and plastid gene has been successfully applied. *Ar. debilis* and its substitute (*Saussurea lappa*) were identified by the ITS sequence^32^; *Solanum lyratum* and its toxic substitute *Ar. mollissima* were identified based on sequences of ITS, *matK*, *rbcL*, *trnH-psbA*, and *trnL-trnF*^33^; TCMs Stephaniae Tetrandrae Radix, Akebiae Caulis, Aucklandia Radix, and Aristolochiae Fructus were identified using the *matK*, *rbcL*, *trnH-psbA*, and *trnL-trnF* DNA regions^34^.

Given that only a limited number of Aristolochiaceous herbs have been previously identified and reported, in this study we comprehensively collected samples of Aristolochiaceous materials and their putative non-Aristolochiaceous substitutes and a sequence library of the standard barcodes ITS2 and *psbA-trnH* was created. In addition, barcode sequence based Real-Time PCR amplification has emerged as a useful tool to detect adulterants. It can be performed in either a singleplex or multiplex mode in combination with high resolution curves or probe hybridization^35^,^36^. Therefore, based on sequence information from ITS2, we show that Real-Time PCR using TaqMan probes can provide an automated analytical and rapid strategy to identify herbs belonging to Aristolochiaceous plants. Lastly, we used Ultra High Performance Liquid Chromatography-Mass Spectrometer (UHPLC-HR-MS) analyses to authentically verify Aristolochiaceous materials containing AAs. Therefore, our results show that using an integrated DNA barcodes, Real-Time PCR with TaqMan probes, and UHPLC-HR-MS can provide an efficient and reliable authentication system for maintaining the therapeutical quality of herbal products and protecting consumers from health risks.

## Results

### Amplification of ITS2 and *psbA-trnH*

Regions of the ITS2 and *psbA-trnH* were amplified from DNA extracted from 289 plant samples (Table 2). ITS2 was successfully amplified from 282 samples (97.60%). From these, 274 samples (97.16%) were successfully sequenced and showed that high-quality bidirectional sequences were obtained. The other 8 samples amplified were used for cloning purposes.

Successful amplification of *psbA-trnH* was obtained from 185 plant samples (63.91%). The lower amplification rate for the universal *psbA-trnH* primers was due to the inability to obtain *psbA-trnH* sequences from the *Asarum* and *Saruma henryi* species. PCR products from 130 samples (70.27%) were successfully sequenced with high-quality bidirectional sequences. However, for the other 55 PCR products, the sequence quality of *psbA-trnH* was poor due to the presence of poly(A/T). Therefore these PCR products were cloned prior to sequencing^37^.

### Sequence Analysis

In this study, 282 ITS2 and 185 *psbA-trnH* sequences were successfully obtained and analyzed from Aristolochiaceous and non-Aristolochiaceous plants (Table 2; additional details in Supplementary Table S2 online). The ITS2 sequences obtained from the different genera of the Aristolochiaceae family, *Aristolochia*, *Asarum*, *Saruma*, and *Thottea*, varied in length from 226 to 280 bp and GC content (47.0–80.2%) (Table 3). ITS2 sequences obtained from the non-Aristolochiaceae family, such as *Cocculus*, *Akebia*, *Clematis*, and *Stachyuru* ranged in length from 181 to 258 bp with GC content ranging from 51.7–73.9% as shown in Table 3.

### Species authentication using ITS2 and *psbA-trnH*

BLAST1, nearest distance, and the neighbor-joining (NJ) tree were used to estimate the capability of authenticating species using ITS2 and *psbA-trnH* as identifying DNA barcodes.

The results showed that the ITS2 and *psbA-trnH* sequences could both be used to successfully identify sample differences at the genus level (100% success rate) when BLAST1 and the nearest distance methods were used. Successful identification of differences at the species level, for ITS2 was 81.8% for BLAST1 and 81.5% for the nearest distance method. For *psbA-trnH*, identification at the species level was 86.2% and 73.0% for BLAST1 and nearest distance method respectively.

**Figure 1:**
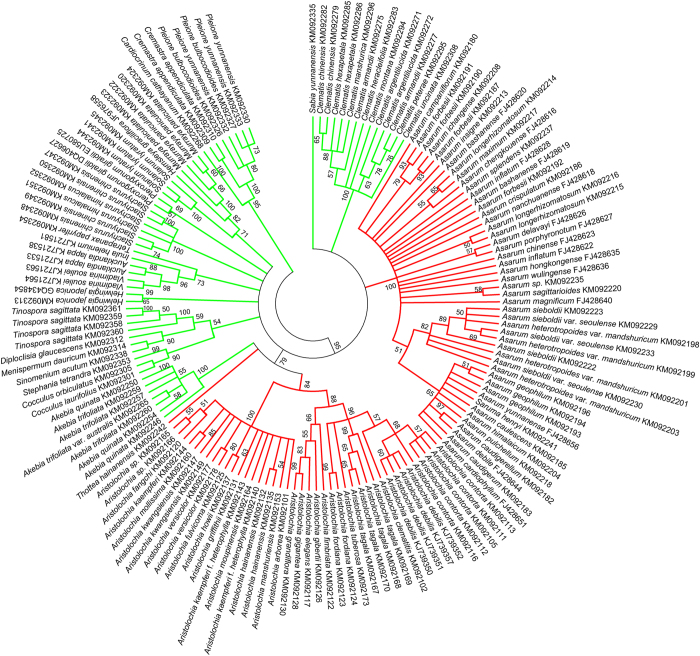
The NJ tree constructed from the ITS2 haplotypes from Aristolochiaceae and non-Aristolochiaceae substitutes. The bootstrap scores (1000 replicates) are shown (≥50%) for each branch.

The NJ tree was also used to authenticate plant species from the Aristolochiaceae family and those from the non-Aristolochiaceous substitutes, using all haplotypes of the ITS2 region (Fig. 1). The NJ tree showed that samples from the Aristolochiaceae family cluster together, whereas non-Aristolochiaceous samples clustered into their own clades. Therefore, the NJ tree and the ITS2 region can be successfully used to distinguish Aristolochiaceous species from their putative substitutes (non-Aristolochiaceae).

All sequences of the *psbA-trnH* region were also used to identify different species using the NJ tree (additional details in Supplementary Fig. S1 online). As for the ITS2 region, the *psbA-trnH* region can also be used to successfully distinguish Aristolochiaceous species from the non-Aristolochiaceous species.

### Rapid detection of Aristolochiaceous plants using Real-Time PCR

**Figure 2:**
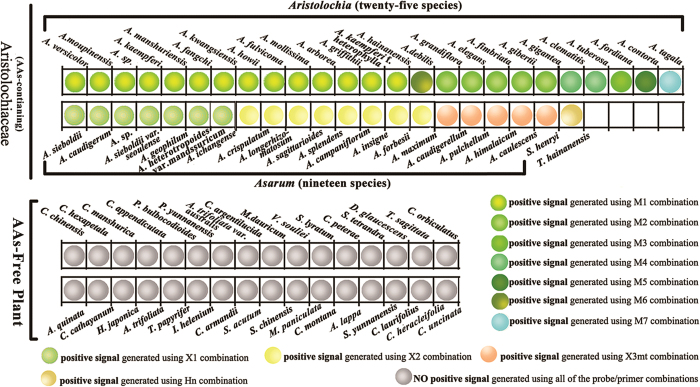
Rapid detection of AAs-containing plants using Real-Time PCR with TaqMan probes.

The Real-Time PCR assays were developed and evaluated using specific primer pairs and TaqMan probes designed from ITS2 sequences obtained from Aristolochiaceous plants. In order to ensure the primer/probe combinations covered all of the collected species of Aristolochiaceous plants and their different genotypes, eleven groups of primer/probe combinations were designed (additional details in Supplementary Table S5 online). All of the primer/probe combinations were specific to the Aristolochiaceae family through the identification of sequences using the non-redundant nucleic acid database (http://blast.st-va.ncbi.nlm.nih.gov/Blast.cgi) and our local herbal medicine database (http://www.tcmbarcode.cn). Additionally, a series of real-time PCR assays were employed to experimentally verify the specificity of all of the primer/probe combinations. The Real-Time PCR results show that all 158 Aristolochiaceous plant samples used in the assay, belonging to 46 species, could generate normal fluorescence amplification curves and Ct values (<35) using their corresponding primer/probe combinations (Fig. 2). In contrast, with the negative control non-Aristolochiaceous plant sample group, either no curves or abnormal amplification curves were generated and the results were thus labeled as “undetermined” (Fig. 2). Therefore, these results show that Real-Time PCR with TaqMan technology, based on the 11 primer/probe combinations, can be successfully used as a rapid method to detect Aristolochiaceous plants.

### The detection of AAs using UHPLC-HR-MS

**Figure 3:**
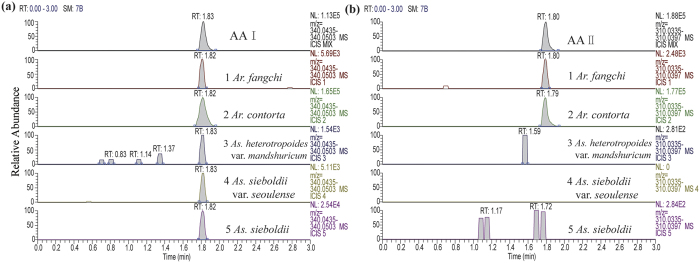
MS profiles of samples 1–5. (**a**) AA I was detected at *m/z* 340.0469 and the standard was AA I. (**b**) AA II was detected at *m/z* 310.0366 and the standard was AA II.

For Aristolochiaceous herbs, the results showed that 39 species contained aristolochic acid I (AA I) (Fig. 3; additional details in Supplementary Table S6 and Fig. S2 online) and 14 species contained aristolochic acid II (AA II) (Fig. 3; additional details in Supplementary Table S6 and Fig. S3 online), and no AAs were found in two *Aristolochia* species (*Ar. kwangsiensis* and *Ar. elegans*) together with four *Asarum* species (*As. caudigerum*, *As. caulescens*, *As. pulchellum*, and *As. campaniflorum*). Our results confirmed that plants from non-Aristolochiaceae family do not contain AA I or II (additional details in Supplementary Table S6 and Figs. S2, S3 online). In this study, AA I and II were determined at *m/z* 340.0469 and 310.0366, respectively (Fig. 3).

### Survey of commercial samples

Out of 28 raw drug samples collected from the market, 19 ITS2 and 11 *psbA-trnH* sequences were obtained from 20 of the samples. For the remaining eight drug samples, no amplification products were obtained even after multiple attempts. This suggests that the DNA in these samples is highly degraded, thus inhibiting amplification and sequencing. Using the sequence information, the reference database and our herbal medicine database (http://www.tcmbarcode.cn), it was found that four raw samples were potentially mislabelled.

The drug sample MDL03, was labelled as Fangji (*Stephania tetrandra*) but was identified in this study as Guangfangji (*Aristolochia fangchi*). Real-Time PCR results showed that MDL03 generated normal fluorescence amplification curves using the M1 primer/probe combinations. UHPLC-HR-MS analysis supports these results as AA I was detected in this sample (additional details in Supplementary Table S7 online).

Another example of misidentification was found for sample MDL06, which contained material from the *Dioscorea sp.* but was labelled as Zhushalian (*Aristolochia tuberosa*). *Dioscorea sp.* has not been included as substitutes in our reference database. For this sample, no amplification curves were generated in the Real-Time PCR assay and no detectable traces of AA I and II were found (additional details in Supplementary Table S7 online).

The other two samples, MDL12 and MDL13, were labelled as Mutong (*Akebia quinata*, *Akebia trifoliate* and *Akebia trifoliate* var. *australis* in Chinese Pharmacopoeia) but were identified as *Clematis* species. Due to the low resolution of ITS2 and *psbA-trnH* for *Clematis,* samples MDL12 to 16 matched to multiple congeneric species. Samples MDL17 to 20 matched to the *Asarum* genus. Real-Time PCR results showed that no amplification curves were obtained from MDL12 to 13 but were obtained from samples MDL17 to 20. UHPLC-HR-MS analysis confirmed that AAs were undetectable in MDL12 and 13. For the four Xixin (*Asarum heterotropoides* var. *mandshuricum*, *Asarum sieboldii* var. *seoulense* and *Asarum sieboldii* in Chinese Pharmacopoeia) samples, AAs were found in MDL17 and 18 but not MDL19 (additional details in Supplementary Table S7 online).

## Discussion

### The ITS2 region is more applicable than psbA-trnH for the identification of Aristolochiaceous plant materials

Statistical analysis showed that limited ITS2 sequences for *Aristolochia* species were available in GenBank. In this study, 87 ITS2 and 87 *psbA-trnH* sequences from 25 *Aristolochia* species were generated and submitted to GenBank (additional details in Supplementary Table S2 online). The NJ tree demonstrated that the ITS2 region can be used to accurately distinguish Aristolochiaceous species from their putative substitutes (non-Aristolochiaceae family). The ITS2 region has been proposed as a universal DNA barcode marker for species identification by Chen *et al.*^24^. Our results highlight that there are more advantages to using the ITS2 sequence than *psbA-trnH*, as it universally covers all species of the Aristolochiaceae family, has a small fragment length ranging from 226 to 280 bp, and amplifies and sequences easily. The *psbA-trnH* sequence could not be obtained from the *Asarum* and *Saruma henryi* species even though two different primer sets were used (additional details in Supplementary Table S3 online)^38^. In addition, some PCR products obtained from the *psbA-trnH* region were difficult to sequence due to the presence of poly(A/T) and molecular cloning was required^37^. Thus, the ITS2 region is a better option than the *psbA-trnH* region for the identification of Aristolochiaceous plant materials.

### DNA barcoding can be used to trace plant materials from Aristolochiceae family

It has been reported that herbal product adulteration and contamination is prevalent in the traditional herbal medicine market place. Consequently, adverse effects of impure materials or ingredients have caused significant effects on the health of some consumers. For example, AAs were detected in Chinese herbal mixtures named “Kidney protection” with no mention of *Aristolochia* materials in the ingredients^39^. In another case, wolly foxglove (*Digitalis lanata* Ehrh.) was misidentified and labeled as plantain resulting in consumers experiencing abnormal heart functions^40^. Accidental or deliberate adulteration and contamination of toxic materials in herbal products presents a considerable health risk for consumers^41^. Identification of dried toxic herbal products based on direct morphology observations is severely hindered by the loss of important characteristics. Although there are a number of chemical standardizations available to identify potential contaminants, they have not been able to trace the identity of adulterants. DNA barcoding and DNA barcode-based approaches have been utilized to identify added adulterants and contaminants in medicinal products^36^,^41^. Li *et al.* (2012) documented that *Solanum lyratum* (Baiying) was replaced with *Ar. mollissima* (Xungufeng) in commodity samples after species identification by DNA barcoding experiments. A similar occurrence was found in this study, where *Stephania tetrandra* (MDL03 sample) was substituted with *Ar. fangchi* from commercial samples obtained from online stores.

This study is the first to use Real-Time PCR arrays with TaqMan probes to detect a wide range of materials from the Aristolochiceae family and from the non-Aristolochiceae substitutes in China. The system described here is accurate, sensitive, and simple-to-use without sequencing process to identify herbal materials in the manufacturing process. In order to establish a specific region to distinguish plants from the Aristolochiceae family from the non-Aristolochiceae samples, we selected the ITS2 loci as the target. Due to relative variability in the ITS2 sequence, every combination of primer and probe detected different groups which contained some species from the Aristolochiceae family, with shared sequence similarity (additional details in Supplementary Table S4 online). A similar strategy was carried out for the identification of *Gentiana macrophylla*^42^. Furthermore, the application has also been successfully used in the detection of a bacterial pathogen and HIV virological failure^43^,^44^.

### Validation of barcoding results using UHPLC-HR-MS technology

Aristolochic acids have been confirmed to induce mutations with A:T to T:A transversions in AA-upper tract urothelial carcinomas patients^45^. AAs are a predominant characteristic of Aristolochiaceous species and can be used as chemical identification markers^46^,^47^. AA I was found to be the most toxic aristolochic acids causing nephrotoxicity, followed by AA II, AA VIIIa, and AA I a in decreasing levels^48^. In order to validate the results from barcoding test, as similarly seen in other relevant comparative studies^49^,^50^,^51^, we collected a broad range of materials, 165 samples from 75 species to analyze AA I and II using UHPLC-HR-MS with the advantages of high sensitivity, high selectivity, and the low detection limitation, most of which corresponded to samples from the DNA barcode experiment. UHPLC-HR-MS analysis confirmed that most of the Aristolochiaceous species contained AA I (86.67%), and 31.11% also contained AA II. Both compounds were not detected in non-Aristolochiaceous samples.

Interestingly, no AAs were detected in four *Asarum* species (*As. caudigerum*, *As. caulescens*, *As. pulchellum*, and *As. campaniflorum*) and two *Aristolochia* species (*Ar. kwangsiensis* and *Ar. elegans*). The possible reasons for this are that the accumulation of secondary metabolites is tightly regulated by environmental factors such as altitude, temperature, humidity and light intensity^52^,^53^. Moreover, different growth stages and years may also play important roles in the synthesis and metabolism of secondary metabolite elements^54^. These ideas could be clarified in the future with studies involving the collection of more Aristolochiaceous plant materials from different ecological environments and/or from various places and growing years. Additionally, UHPLC-HR-MS technology is a complementary method that can be used when DNA barcodes are difficult to obtain due to DNA degradation in long-term preserved and processed materials^55^.

## Methods

### Candidate barcodes and plant materials

In this research, the applicability of one nuclear (ITS2) and one plastid (*psbA-trnH*) loci were selected as candidate barcodes. The sampling included 289 accessions from 79 species belonging to 24 genera, distributed in 13 families (Table 2). Within the Aristolochiaceae family, 158 samples from 46 species belonging to the four genera (*Aristolochia*, *Asarum*, *Saruma*, and *Thottea*) were obtained. 33 non-Aristolochiaceous herb species (often used as possible substitutes for Aristolochiaceous herbs) were also sampled and contained 20 genera from 12 families. 45 species of Aristolochiaceous plants and their putative substitutes were further divided into seven groups referring to Fangji, Mutong, Qingmuxiang, Baimaoteng, Zhushalian, Madouling, and Shancigu according to the same or similar domestic Chinese name in every group (additional details in Supplementary Table S2 online). All the samples were morphologically identified by Wei Sun at the Institute of Chinese Materia Medica, China Academy of Chinese Medical Sciences. All voucher samples were stored in the Herbarium of the Institute of Chinese Materia Medica, China Academy of Chinese Medical Sciences and the School of Pharmaceutical Sciences, Peking University Health Science Centre, Beijing, China. DNA extracted from *Ar. clematitis* was kindly provided by the Royal Botanical Garden, Kew, UK. In addition, 15 ITS2 sequences from 13 *Asarum* species, two ITS2 sequences of *Pteroxygonum giraldii*, four ITS2 and four *psbA-trnH* sequences of *Hemsleya graciliflora* were downloaded from GenBank (additional details in Supplementary Table S2 online). In this study, we tried to cover all genera referring to known and suspected herbs containing AAs based on FDA safety alerts (http://www.fda.gov/Food/RecallsOutbreaksEmergencies/SafetyAlertsAdvisories/ucm095283.htm).

To validate the integrated method for testing commodity samples from the market, twenty eight herbal samples labelled with the vernacular names Fangji, Mutong, Chuanmutong, Zhushalian, Xungufeng, Baiying or Xixin in Chinese were collected from different raw drug retail markets or online stores. The collected samples were deposited in the Institute of Chinese Materia Medica, China Academy of Chinese Medical Sciences. The commercial samples details are shown in Supplementary Table S7. Identification of the raw materials was conducted by comparing Basic Local Alignment Search Tool (BLAST) data against our reference data with best-hit match.

### DNA Extraction, PCR amplification, and sequencing

Genomic DNA was isolated from 30–40 mg dried material using the Plant Genomic DNA Kit (Tiangen Biotech Co., China), according to the DNA extraction protocol. The ITS2 and *psbA-trnH* regions were amplified using universal primers and general PCR reaction conditions (additional details in Supplementary Table S3 online)^24^,^56^. PCR amplification was performed in 25 μL reaction mixtures containing 20–50 ng of genomic DNA template, 2 × Taq PCR MasterMix (Beijing Aidlab Biotech Co., China), 1 μL of each primer (2.5 μM) and distilled deionized water. The majority of PCR products were purified and directly sequenced using the same primers as for the PCR in an ABI 3730 XL sequencer (SinoGenoMax Co.,Ltd.). Other PCR products were purified using the DNA-gel Purification Kit (Tiangen Biotech Co., China), cloned in competent *Escherichia coli* cells (strain DH5α) using pMD^TM^19-T Vector System (Takara Biotech, China) and then sequenced by 3730 XL sequencer.

### Sequence analysis

The CodonCode Aligner 4.0.4 (CodonCode Co., USA) was used to proofread, assemble the contigs, and generate consensus sequences. Based on the HMMer software, the ITS2 region was obtained by function of annotation^57^,^58^. The alignment was performed with Clustal W. The tree-based method was used for species identification analyses: the neighbor-joining (NJ) tree was conducted by MEGA 5.0 with 1000 bootstrap replicates^59^. The bootstrap value above 50% is shown. Two methods of species identification, namely the Basic Local Alignment Search Tool 1 (BLAST1) and the nearest distance method, were performed to evaluate species identification efficiency^24^,^60^.

### Rapid detection of Aristolochiaceous plants using TaqMan probe technology

The TaqMan Real-time PCR assays were used to rapidly detect Aristolochiaceous plants in this study. The ITS2 sequences obtained from Aristolochiaceous plants were divided into 11 groups according to their homology (additional details in Supplementary Table S4 online). Primers and probes were designed in the conserved region for each group using Primer Express v3.0 and primer 3 (http://bioinfo.ut.ee/ primer3-0.4.0/). The specificity of each primer pair and probes were additionally assessed based on the non-redundant database at NCBI (http://blast.st-va.ncbi.nlm.nih.gov/Blast.cgi) and our herbal medicine library (http://www.tcmbarcode.cn) with BLAST algorithm. The primers and probes used in this assay were synthesized by Invitrogen Trading Co., Ltd. (Shanghai) and listed in Supplementary Table S5. Quantitative real-time PCR was performed with three technical replicates per sample using GoldStar TaqMan Mixture with ROX, 1 μL DNA, 0.2 μM specific probe and 0.2 μM of each primer in a 50 μL reaction system as per manufacturer’s instructions (CoWin Bioscience, China) on an ABI 7500 real-time PCR system (Applied Biosystem, USA). All of the Aristolochiaceous plants collected (Table 2) were used as positive samples to verify the in-silico-determined specificity of the primer/probe combinations. In addition, DNA from 60 non-Aristolochiaceous samples belonging to 33 species (Table 2), which shared common names with the Aristolochiaceous plants, were used as negative controls. DNA quality was assessed by classical PCR using ITS2 universal primers. Data collected was analyzed using the ABI 7500 Software (Applied Biosystem, USA), and the quality of the data obtained was determined by fluorescence amplification curve and threshold cycle (Ct) value.

### UHPLC-HR-MS analyses

In this study, 45 species from Aristolochiaceae family and 30 species of non-Aristolochiaceae family were detected by UHPLC-HR-MS. Unfortunately, *Ar. clematitis*, one of the *Aristolochia* species, and three non-Aristolochiaceous species (*Clematis heracleifolia*, *Cl. uncinata*, and *Stachyurus himalaicus*) were undetected due to lack of materials.

Standards of AA I and AA II were purchased from the National Institutes for Food and Drug Control (Beijing, China) and the Research Center of Zhongxin Pharmaceuticals (Tianjin, China), respectively. The purity of all compounds was greater than 98% (determined by HPLC). Acetonitrile and formic acid (HPLC grade) for UPLC analysis were bought from Fisher (Fair Lawn, New Jersey, USA). Deionized water was obtained from Millipore Milli-Q purification system (Millipore, Bedford, MA, USA). The powders for each sample were precisely weighed out to 0.1 g and extracted with 2.5 mL methanol using the ultrasonic method at room temperature for 40 min. The extracts were filtered through a 0.22 μm filter, and 1 μl of filtrate was directly used for UHPLC-HR-MS analysis.

LC analyses were performed with an UltiMate 3000 UHPLC System (Thermo-Fisher Scientific, Germany) equipped with an autosampler, a quaternary pump, a vacuum degasser, a thermostated column compartment and a diode array detector (DAD). Sample separation was performed on a C_18_ column (Bds Hypersil C_18_, 2.4 μm, 150 × 2.1 mm) using a gradient solvent system comprised of 0.1% formic acid in water (A) and acetonitrile (B): 0 −0.3 min, 10% B to 100% B; 0.3 −4 min, 100% B to 100% B; flow rate, 0.3 ml/min.

The LC system was connected to an LTQ Orbitrap Velos pro mass spectrometer (Thermo-Fisher Scientific, Germany) with an electrospray ionization (ESI) source. The ESI source was operated in the negative mode under the following specific conditions: Spray Voltage, 5.0 kV; Sheath Gas Flow Rate, 35 arbitrary units; capillary temperature, 350 ^o^C. Nitrogen (>99.98%) was used as sheath gas. The scan type was a full scan event (*m/z* range 200–500) at resolution of 30000. Mixtures of AA I and II were used as standards. Instrument control and data acquisition was performed with Xcalibur 2.2 software (Thermo-Fisher Scientific, Germany). An external calibration for mass accuracy was carried out the day before the analysis according to the manufacturer’s guidelines.

## Additional Information

**How to cite this article**: Wu, L. *et al.* An integrated system for identifying the hidden assassins in traditional medicines containing aristolochic acids. *Sci. Rep.* **5**, 11318; doi: 10.1038/srep11318 (2015).

## Supplementary Material

Supplementary Information

## Figures and Tables

**Table 1:**
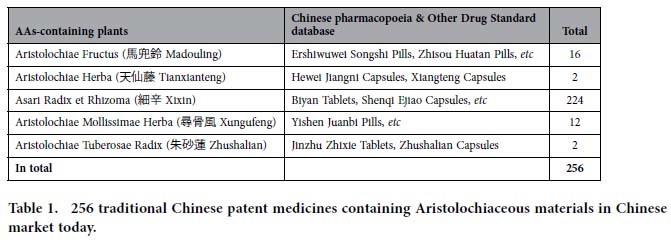
256 traditional Chinese patent medicines containing Aristolochiaceous materials in Chinese market today.

**Table 2:** Aristolochiaceae and non-Aristolochiaceae samples and sequence information.

	**Genus**	**species number**	**Sample**	**ITS2**	***psbA-trnH***
**Aristolochiaceae**(AAs-Containing plants)	*Aristolochia*	25	91	87	87
	*Asarum*	19	61	61	/
	*Saruma*	1	1	1	/
	*Thottea*	1	5	4	5
**Non-Aristolochiaceae** (AAs-Free plants)	*Clematis*, *Cocculus,* *Akebia*, *etc*. 20 genera from 12 families⋇	33	131	129	93
**Total**		79	289	282	185

The detail information can be found in Supplementary Table S2.

^⋇^The plants share common TCM names with some of the Aristolochiaceae species and are difficult to distinguish by their morphological features, e.g. Mutong, Fangji, Qingmuxiang, Baimaoteng, Zhushalian, Shancigu, Madouling. / indicated failed to obtain.

**Table 3:** Sequence analysis of ITS2 and *psbA-trnH* from Aristolochiaceae and non-Aristolochiaceae plants.

	**genus**	**ITS2**		***psbA-trnH***	
		**Sequence length**	**GC content (mean)%**	**Sequence length**	**GC content (mean)%**
**Aristolochiaceae**	*Aristolochia*	256–280	66.7–80.2(74.7)	215–297	34.0–42.2(39.2)
	*Asarum*	226–234	49.8–56.4(53.0)	/	/
	*Saruma henryi*	234	47.0	/	/
	*Thottea hainanensis*	273	76.9	219–223	39.0–42.0(40.3)
**non-Aristolochiaceae**	20 genera	181–258	51.7–73.9(65.2)	230–898	21.7–40.0(29.5)

/failed to obtain.
