# Supplementary material for: An integrated system for identifying the hidden assassins in traditional medicines containing aristolochic acids
Source: Sci Rep. 2015 Aug 13;5:11318. doi: 10.1038/srep11318 (PMC4535178; doi:10.1038/srep11318)
Supplement: Supplementary Information [file srep11318-s1.pdf]

TITLE: An integrated system for identifying the hidden assassins in traditional medicines containing aristolochic acids

AUTHORS: Lan Wu[1,2 §], Wei Sun[1 §], Bo Wang[3], Haiyu Zhao[1], Yaoli Li[4], Shaoqing Cai[4], Li Xiang[1], Yingjie Zhu[1], Hui Yao[3], Jingyuan Song[3], Yung-Chi Cheng[5], Shilin Chen[1*]

SUPPLEMENTARY INFORMATION

**Supplementary Table S1.** A detailed list of 256 traditional Chinese patent medicines containing Aristolochiaceous materials in Chinese market today.

**Supplementary Table S2.** Species information for Aristolochiaceous and non-Aristolochiaceous plants used in this study.

**Supplementary Table S3.** List of universal primers and reaction conditions for ITS2 and *psbA-trnH*.

**Supplementary Table S4.** List of the 11 groups based on ITS2 sequence similarity from Aristolochiaceae.

**Supplementary Table S5.** Primers and Hydrolysis Probes used in Aristolochiaceous plants detection.

**Supplementary Table S6.** Detection of AA Ⅰ and Ⅱ for all species using UHPLC-HR-MS analysis.

**Supplementary Table S7.** Summary of commercial samples identified by DNA Barcoding, Real-Time PCR and UHPLC-HR-MS.

**Supplementary Fig. S1.** The NJ tree constructed from the *psbA-trnH* region from Aristolochiaceae and non-Aristolochiaceae substitutes. The bootstrap scores (1000 replicates) are shown (≥50%) for each branch.

**Supplementary Fig. S2.** MS profiles of AA Ⅰ for all species in UHPLC-HR-MS analysis. AA Ⅰ was detected at *m/z* 340.0469.

**Supplementary Fig. S3.** MS profiles of AA Ⅱ for all species in UHPLC-HR-MS analysis. AA Ⅱ was detected at *m/z* 310.0366.

**Table S1. A detailed list of 256 traditional Chinese patent medicines containing Aristolochiaceous materials in Chinese market today.**

| No. | AAs-containing plants | Traditional Chinese patent medicines name | Drug Standard database |
|---|---|---|---|
| 1 | Aristolochiae Fructus | Siwei Zhixiemutang Powder | MOH |
| 2 | Aristolochiae Fructus | Zhisou Huatan Capsules | MOH |
| 3 | Aristolochiae Fructus | Ershiwuwei Songshi Pills | Ch.P, 2000 edition |
| 4 | Aristolochiae Fructus | Zhisou Huatan Pills | Ch.P, 2000 edition |
| 5 | Aristolochiae Fructus | Xin Bitaoxian Tablets | MOH (volume 2) |
| 6 | Aristolochiae Fructus | Jingzhi Kesou Tanchuan Pills[a] | MOH (volume 20) |
| 7 | Aristolochiae Fructus | Zhisou Qingguo Tablets | MOH (volume 20) |
| 8 | Aristolochiae Fructus | Weifu Granules | MOH (volume 18) |
| 9 | Aristolochiae Fructus | Chuanxiling Capsules | MOH (volume 15) |
| 10 | Aristolochiae Fructus | Feian Tablets | MOH (volume 15) |
| 11 | Aristolochiae Fructus | Fufang Shedan Chuanbei Powder | MOH (volume 11) |
| 12 | Aristolochiae Fructus | Ershiwei Shugan Capsules | CFDA |
| 13 | Aristolochiae Fructus | Ershiwuwei Lüronghao Capsules | CFDA |
| 14 | Aristolochiae Fructus | Shisanwei Shugan Capsules | CFDA |
| 15 | Aristolochiae Fructus | Shisiwei Shugan Capsules | CFDA |
| 16 | Aristolochiae Fructus | Sishierwei Shugan Capsules | CFDA |
| 17 | Aristolochiae Herba | Hewei Jiangni Capsules | CFDA |
| 18 | Aristolochiae Herba | Xiangteng Capsules | CFDA |
| 19 | Asari Radix et Rhizoma | Shenqi Capsules | MOH |
| 20 | Asari Radix et Rhizoma | Shenqi Granules | MOH |
| 21 | Asari Radix et Rhizoma | Jiuwei Qianghuo Oral Liquid | MOH |
| 22 | Asari Radix et Rhizoma | Xiaoqinglong Capsules | MOH |
| 23 | Asari Radix et Rhizoma | Fufang Nanxing Zhitong Plaster | MOH |
| 24 | Asari Radix et Rhizoma | Fufang Weiling Plaster | MOH |
| 25 | Asari Radix et Rhizoma | Yitongshu Oral Liquid | MOH |
| 26 | Asari Radix et Rhizoma | Jinguan Tablets | CFDA |
| 27 | Asari Radix et Rhizoma | Qingshi Granule | CFDA |
| 28 | Asari Radix et Rhizoma | Zhennaoning Capsules | MOH |
| 29 | Asari Radix et Rhizoma | Biyuanshu Capsules | CFDA |
| 30 | Asari Radix et Rhizoma | Danguixiang Granules | CFDA |
| 31 | Asari Radix et Rhizoma | Tongtian Oral Liquid | CFDA |
| 32 | Asari Radix et Rhizoma | Xiaoshangtong Liniment | CFDA |
| 33 | Asari Radix et Rhizoma | Yangxue Qingnao Granules | CFDA |
| 34 | Asari Radix et Rhizoma | Xiaoqinglong Granule | MOH |
| 35 | Asari Radix et Rhizoma | Tongbi Kanggan Mixture | CFDA |
| 36 | Asari Radix et Rhizoma | Shenqi Tablets | MOH |
| 37 | Asari Radix et Rhizoma | Biyan Tablets | Ch.P, 2000 edition |

| | | | |
|---|---|---|---|
| 38 | Asari Radix et Rhizoma | Chuanxiong Chatiao Powder | Ch.P, 2000 edition |
| 39 | Asari Radix et Rhizoma | Chuanxiong Chatiao Pills | Ch.P, 2000 edition |
| 40 | Asari Radix et Rhizoma | Ertong Qingfei Pills | Ch.P, 2000 edition |
| 41 | Asari Radix et Rhizoma | Jiuwei Qianghuo Granules | Ch.P, 2000 edition |
| 42 | Asari Radix et Rhizoma | Jiuwei Qianghuo Pills | Ch.P, 2000 edition |
| 43 | Asari Radix et Rhizoma | Shuzheng Tablets | Ch.P, 2000 edition |
| 44 | Asari Radix et Rhizoma | Tongguan Powder | Ch.P, 2000 edition |
| 45 | Asari Radix et Rhizoma | Xiaoqinglong Mixture | Ch.P, 2000 edition |
| 46 | Asari Radix et Rhizoma | Xiaoqinglong Granules | Ch.P, 2000 edition |
| 47 | Asari Radix et Rhizoma | Zaizao Pills | Ch.P, 2000 edition |
| 48 | Asari Radix et Rhizoma | Lusiluo Pills | Ch.P, 2000 edition |
| 49 | Asari Radix et Rhizoma | Dongfang Huoxue Plaster | MOH (volume 16) |
| 50 | Asari Radix et Rhizoma | Hanshibi Granules | MOH (volume 16) |
| 51 | Asari Radix et Rhizoma | Hanshibi Tablets | MOH (volume 16) |
| 52 | Asari Radix et Rhizoma | Kelisha Capsules | MOH (volume 16) |
| 53 | Asari Radix et Rhizoma | Duhuo Jisheng Mixture | MOH (volume 8) |
| 54 | Asari Radix et Rhizoma | Huisheng Zaizao Pills | MOH (volume 8) |
| 55 | Asari Radix et Rhizoma | Biyanling Tablets | MOH (volume 2) |
| 56 | Asari Radix et Rhizoma | Chitongning | MOH (volume 2) |
| 57 | Asari Radix et Rhizoma | Duhuo Jisheng Pills | MOH (volume 2) |
| 58 | Asari Radix et Rhizoma | Fengshang Zhitong Plaster | MOH (volume 2) |
| 59 | Asari Radix et Rhizoma | Huoxue Zhitong Plaster | MOH (volume 2) |
| 60 | Asari Radix et Rhizoma | Jisheng Zhuifeng Vinum | MOH (volume 2) |
| 61 | Asari Radix et Rhizoma | Tianma Zhuifeng Plaster | MOH (volume 2) |
| 62 | Asari Radix et Rhizoma | Wanying Baozhen Plaster | MOH (volume 2) |
| 63 | Asari Radix et Rhizoma | Xuanyao Yuhong Ointment | MOH (volume 2) |
| 64 | Asari Radix et Rhizoma | Yatong Water | MOH (volume 2) |
| 65 | Asari Radix et Rhizoma | Zhitong Water | MOH (volume 2) |
| 66 | Asari Radix et Rhizoma | Chuanxiong Chatiao Oral Liquid | MOH (volume 20) |
| 67 | Asari Radix et Rhizoma | Fengshi Antai Tablets | MOH (volume 20) |
| 68 | Asari Radix et Rhizoma | Foshan Renshen Zaizao Tablets | MOH (volume 20) |
| 69 | Asari Radix et Rhizoma | Keyangmin Spirit | MOH (volume 20) |
| 70 | Asari Radix et Rhizoma | Liujing Toutong Tablets | MOH (volume 20) |
| 71 | Asari Radix et Rhizoma | Wudidan Capsules | MOH (volume 20) |
| 72 | Asari Radix et Rhizoma | Xiaoer Zhixiaoling Tablets | MOH (volume 20) |
| 73 | Asari Radix et Rhizoma | Xiaoqinglong Oral Liquid | MOH (volume 20) |
| 74 | Asari Radix et Rhizoma | Xiaoqinglong Syrup | MOH (volume 20) |
| 75 | Asari Radix et Rhizoma | Quanzhou Baicaoqu | MOH (volume 9) |
| 76 | Asari Radix et Rhizoma | Yanxianfengdian Pills | MOH (volume 9) |
| 77 | Asari Radix et Rhizoma | Fahan Jiere Pills | MOH (volume 6) |

| | | | |
|---|---|---|---|
| 78 | Asari Radix et Rhizoma | Fengtong Pills | MOH (volume 6) |
| 79 | Asari Radix et Rhizoma | Juhua Chatiao Powder | MOH (volume 6) |
| 80 | Asari Radix et Rhizoma | Mingmu Yanggan Pills | MOH (volume 6) |
| 81 | Asari Radix et Rhizoma | Ruyi Oil | MOH (volume 6) |
| 82 | Asari Radix et Rhizoma | Shaoshang Spray | MOH (volume 6) |
| 83 | Asari Radix et Rhizoma | Shulere Sticking Plaster | MOH (volume 6) |
| 84 | Asari Radix et Rhizoma | Tougu Zhenfeng Pills | MOH (volume 6) |
| 85 | Asari Radix et Rhizoma | Wanling Tablets | MOH (volume 6) |
| 86 | Asari Radix et Rhizoma | Weibing Pills | MOH (volume 6) |
| 87 | Asari Radix et Rhizoma | Lucixian Pills | MOH (volume 6) |
| 88 | Asari Radix et Rhizoma | Baoan Wanning Pills | MOH (volume 7) |
| 89 | Asari Radix et Rhizoma | Chuanxiong Chatiao Teabag | MOH (volume 7) |
| 90 | Asari Radix et Rhizoma | Guanjie Zhentong Plaster | MOH (volume 7) |
| 91 | Asari Radix et Rhizoma | Jiedu Wanling Pills | MOH (volume 7) |
| 92 | Asari Radix et Rhizoma | Kechuan Ointment | MOH (volume 7) |
| 93 | Asari Radix et Rhizoma | Pinggan Shuluo Pills | MOH (volume 7) |
| 94 | Asari Radix et Rhizoma | Shufeng Zaizao Pills | MOH (volume 7) |
| 95 | Asari Radix et Rhizoma | Xinqin Granule | MOH (volume 7) |
| 96 | Asari Radix et Rhizoma | Yaojiu Pills | MOH (volume 7) |
| 97 | Asari Radix et Rhizoma | Zhenggu Plaster | MOH (volume 7) |
| 98 | Asari Radix et Rhizoma | Anyang Hugu Vinum | MOH (volume 3) |
| 99 | Asari Radix et Rhizoma | Babao Zhenjing Pills | MOH (volume 3) |
| 100 | Asari Radix et Rhizoma | Baihuashe Plaster | MOH (volume 3) |
| 101 | Asari Radix et Rhizoma | Dieda Fengshi Vinum | MOH (volume 3) |
| 102 | Asari Radix et Rhizoma | Fufang Banxia Tablets | MOH (volume 3) |
| 103 | Asari Radix et Rhizoma | Hanchuan Pills | MOH (volume 3) |
| 104 | Asari Radix et Rhizoma | Hanshibi Pills | MOH (volume 3) |
| 105 | Asari Radix et Rhizoma | Kangshuan Zaizao Pills | MOH (volume 3) |
| 106 | Asari Radix et Rhizoma | Biyuanshu Oral Liquid | MOH (volume 18) |
| 107 | Asari Radix et Rhizoma | Dieda Wanhua Oil | MOH (volume 18) |
| 108 | Asari Radix et Rhizoma | Duji Dujiao Plaster | MOH (volume 18) |
| 109 | Asari Radix et Rhizoma | Huanjingjian Oral Liquid | MOH (volume 18) |
| 110 | Asari Radix et Rhizoma | Tezhi Dogskin Plaster | MOH (volume 18) |
| 111 | Asari Radix et Rhizoma | Tianhe Zhuifeng Plaster | MOH (volume 18) |
| 112 | Asari Radix et Rhizoma | Yunxiangjing | MOH (volume 18) |
| 113 | Asari Radix et Rhizoma | Zhuifeng Tougu Pills | MOH (volume 18) |
| 114 | Asari Radix et Rhizoma | Biyanling Capsules | MOH (volume 10) |
| 115 | Asari Radix et Rhizoma | Shensanqi Shangyao | MOH (volume 10) |
| 116 | Asari Radix et Rhizoma | Chanling Pills | MOH (volume 10) |
| 117 | Asari Radix et Rhizoma | Chenxiang Powder | MOH (volume 10) |

| | | | |
|---|---|---|---|
| 118 | Asari Radix et Rhizoma | Chitong Xiaoyanling Granule | MOH (volume 10) |
| 119 | Asari Radix et Rhizoma | Guanjie Jietong Plaster | MOH (volume 10) |
| 120 | Asari Radix et Rhizoma | Houzao Niuhuang Powder | MOH (volume 10) |
| 121 | Asari Radix et Rhizoma | Jinguning Liniment | MOH (volume 10) |
| 122 | Asari Radix et Rhizoma | Xiaoer Feibining Tablets | MOH (volume 10) |
| 123 | Asari Radix et Rhizoma | Xiaoer Kechuan Granule | MOH (volume 10) |
| 124 | Asari Radix et Rhizoma | Kuanxiong Aerosol | MOH (volume 12) |
| 125 | Asari Radix et Rhizoma | Qingyu Biwen Dan | MOH (volume 12) |
| 126 | Asari Radix et Rhizoma | Tuizhang Ophthalmic Ointment | MOH (volume 12) |
| 127 | Asari Radix et Rhizoma | Xiaozhong Zhitong Tincture | MOH (volume 12) |
| 128 | Asari Radix et Rhizoma | Yulong Oil | MOH (volume 12) |
| 129 | Asari Radix et Rhizoma | Zhuifeng Zhuanggu Plaster | MOH (volume 12) |
| 130 | Asari Radix et Rhizoma | Chuanxiong Chatiao Pills (concentrated pills) | MOH (volume 19) |
| 131 | Asari Radix et Rhizoma | Houyan Pills | MOH (volume 19) |
| 132 | Asari Radix et Rhizoma | Shangyou Liniment | MOH (volume 19) |
| 133 | Asari Radix et Rhizoma | Wudi Vinum | MOH (volume 19) |
| 134 | Asari Radix et Rhizoma | Wudi Zhitong Liniment | MOH (volume 19) |
| 135 | Asari Radix et Rhizoma | Zhanjiang Sheyao | MOH (volume 19) |
| 136 | Asari Radix et Rhizoma | Zhike Huatan Pills | MOH (volume 19) |
| 137 | Asari Radix et Rhizoma | Foshan Renshen Zaizao Pills | MOH (volume 17) |
| 138 | Asari Radix et Rhizoma | Shangshi Baozhen Plaster | MOH (volume 17) |
| 139 | Asari Radix et Rhizoma | Tiaogu Tablets | MOH (volume 17) |
| 140 | Asari Radix et Rhizoma | Tongren Dahuoluo Pills | MOH (volume 17) |
| 141 | Asari Radix et Rhizoma | Waiyong Wudi Plaster | MOH (volume 17) |
| 142 | Asari Radix et Rhizoma | Wuyan Moxa Stick Moxibustion | MOH (volume 17) |
| 143 | Asari Radix et Rhizoma | Yitongshu Injection | MOH (volume 17) |
| 144 | Asari Radix et Rhizoma | Zhengtian Pills | MOH (volume 17) |
| 145 | Asari Radix et Rhizoma | Zhuangyuanhong Vinum | MOH (volume 17) |
| 146 | Asari Radix et Rhizoma | Baicao Oil | MOH (volume 13) |
| 147 | Asari Radix et Rhizoma | Ganteling Capsules | MOH (volume 13) |
| 148 | Asari Radix et Rhizoma | Wenzhong Zhixie Pills | MOH (volume 13) |
| 149 | Asari Radix et Rhizoma | Xinwuyou Tablets | MOH (volume 13) |
| 150 | Asari Radix et Rhizoma | Zhenjing Powder | MOH (volume 13) |
| 151 | Asari Radix et Rhizoma | Zhongfeng Zaizao Pills | MOH (volume 13) |
| 152 | Asari Radix et Rhizoma | Guzengsheng Zhentong Plaster | MOH (volume 14) |
| 153 | Asari Radix et Rhizoma | Mahuang Zhisou Pills | MOH (volume 14) |
| 154 | Asari Radix et Rhizoma | Tongluo Huoxue Pills | MOH (volume 14) |
| 155 | Asari Radix et Rhizoma | Qishe Zhuifeng Vinum | MOH (volume 14) |
| 156 | Asari Radix et Rhizoma | Shensanqi Shangyao Powder | MOH (volume 15) |

| | | | |
|---|---|---|---|
| 157 | Asari Radix et Rhizoma | Shenshe Huoluo Pills | MOH (volume 15) |
| 158 | Asari Radix et Rhizoma | Kelisha Pettets | MOH (volume 15) |
| 159 | Asari Radix et Rhizoma | Ketong Tincture | MOH (volume 15) |
| 160 | Asari Radix et Rhizoma | Renshen Zaizao Pills (concentrated pills) | MOH (volume 15) |
| 161 | Asari Radix et Rhizoma | Sanyu Shenqu | MOH (volume 15) |
| 162 | Asari Radix et Rhizoma | Shangke Zhuanggu Plaster | MOH (volume 15) |
| 163 | Asari Radix et Rhizoma | Shangtongshu | MOH (volume 15) |
| 164 | Asari Radix et Rhizoma | Toufeng Plaster | MOH (volume 15) |
| 165 | Asari Radix et Rhizoma | Xinfang Biyan Capsules | MOH (volume 15) |
| 166 | Asari Radix et Rhizoma | Shaqi Pills | MOH (volume 15) |
| 167 | Asari Radix et Rhizoma | Baxian Oil | MOH (volume 11) |
| 168 | Asari Radix et Rhizoma | Bogu Water | MOH (volume 11) |
| 169 | Asari Radix et Rhizoma | Jieshu Tablets | MOH (volume 11) |
| 170 | Asari Radix et Rhizoma | Jiulong Huafeng Pills | MOH (volume 11) |
| 171 | Asari Radix et Rhizoma | Qufeng Suhe Pills | MOH (volume 11) |
| 172 | Asari Radix et Rhizoma | Tanchuan Banxia Granules | MOH (volume 11) |
| 173 | Asari Radix et Rhizoma | Zhuifeng Tougu Tablets | MOH (volume 11) |
| 174 | Asari Radix et Rhizoma | Shenrong Mugua Vinum | MOH (volume 4) |
| 175 | Asari Radix et Rhizoma | Shensanqi Shangyao Pills | MOH (volume 4) |
| 176 | Asari Radix et Rhizoma | Huitian Zaizao Pills | MOH (volume 4) |
| 177 | Asari Radix et Rhizoma | Jingfeng Pills | MOH (volume 4) |
| 178 | Asari Radix et Rhizoma | Libi Tablets | MOH (volume 4) |
| 179 | Asari Radix et Rhizoma | Shangtongning Tablets | MOH (volume 4) |
| 180 | Asari Radix et Rhizoma | Tongqiao Powder | MOH (volume 4) |
| 181 | Asari Radix et Rhizoma | Weiyanning Granule | MOH (volume 4) |
| 182 | Asari Radix et Rhizoma | Xiaochuan Plaster | MOH (volume 4) |
| 183 | Asari Radix et Rhizoma | Xingnao Zaizao Capsules | MOH (volume 4) |
| 184 | Asari Radix et Rhizoma | Xingnao Zaizao Pills | MOH (volume 4) |
| 185 | Asari Radix et Rhizoma | Biwen Tablets | MOH (volume 5) |
| 186 | Asari Radix et Rhizoma | Chuanxiong Chatiao Granule | MOH (volume 5) |
| 187 | Asari Radix et Rhizoma | Chuanxiong Chatiao Tablets | MOH (volume 5) |
| 188 | Asari Radix et Rhizoma | Ditong Biyan Water | MOH (volume 5) |
| 189 | Asari Radix et Rhizoma | Fugui Fengshi Plaster | MOH (volume 5) |
| 190 | Asari Radix et Rhizoma | Jiebiao Zhuifeng Pills | MOH (volume 5) |
| 191 | Asari Radix et Rhizoma | Jiuwei Qianghuo Granules | MOH (volume 5) |
| 192 | Asari Radix et Rhizoma | Jiuwei Qianghuo Tablets | MOH (volume 5) |
| 193 | Asari Radix et Rhizoma | Eryi Pills | MOH (volume 1) |
| 194 | Asari Radix et Rhizoma | Fengshi Vinum | MOH (volume 1) |
| 195 | Asari Radix et Rhizoma | Huoluo Pills | MOH (volume 1) |

| | | | |
|---|---|---|---|
| 196 | Asari Radix et Rhizoma | Sheyao Pills | MOH (volume 1) |
| 197 | Asari Radix et Rhizoma | Xiaoer Baoan Pills | MOH (volume 1) |
| 198 | Asari Radix et Rhizoma | Baozhen Adhesive Plaster | CFDA |
| 199 | Asari Radix et Rhizoma | Bitong Sticking Plaster | CFDA |
| 200 | Asari Radix et Rhizoma | Shenbei Kechuan Pills | CFDA |
| 201 | Asari Radix et Rhizoma | Cangxin Aerosol | CFDA |
| 202 | Asari Radix et Rhizoma | Chenxiang Anshen Capsules | CFDA |
| 203 | Asari Radix et Rhizoma | Chuanhua Zhitong Odynolysis Plastics | CFDA |
| 204 | Asari Radix et Rhizoma | Chuanyu Fenghan Sticking Plaster | CFDA |
| 205 | Asari Radix et Rhizoma | Dahuoluo Pills | CFDA |
| 206 | Asari Radix et Rhizoma | Dange Jingshu Capsules | CFDA |
| 207 | Asari Radix et Rhizoma | Danzhen Toutong Capsules | CFDA |
| 208 | Asari Radix et Rhizoma | Dingkun Dan | CFDA |
| 209 | Asari Radix et Rhizoma | Fengshi Zhuifeng Plaster | CFDA |
| 210 | Asari Radix et Rhizoma | Fufang Ganmao Capsules | CFDA |
| 211 | Asari Radix et Rhizoma | Fufang Ganmao Tablets | CFDA |
| 212 | Asari Radix et Rhizoma | Fufang Zhuifeng Plaster | CFDA |
| 213 | Asari Radix et Rhizoma | Fufang Zhizi Aerosol | CFDA |
| 214 | Asari Radix et Rhizoma | Ganteling Tablets | CFDA |
| 215 | Asari Radix et Rhizoma | Guwei Huoluo Tincture | CFDA |
| 216 | Asari Radix et Rhizoma | Gushen Buqi Powder | CFDA |
| 217 | Asari Radix et Rhizoma | Guanjie Zhentong Cataplasm | CFDA |
| 218 | Asari Radix et Rhizoma | Jiedu Liyan Pills | CFDA |
| 219 | Asari Radix et Rhizoma | Jingu Dieda Pills | CFDA |
| 220 | Asari Radix et Rhizoma | Jiuji Xingjun Capsules | CFDA |
| 221 | Asari Radix et Rhizoma | Jiuji Xingjun Powder | CFDA |
| 222 | Asari Radix et Rhizoma | Qianshan Huoxue Plaster | CFDA |
| 223 | Asari Radix et Rhizoma | Renshen Zaizao Pills | CFDA |
| 224 | Asari Radix et Rhizoma | Sanbian Wenyang Capsules | CFDA |
| 225 | Asari Radix et Rhizoma | Sanfeng Huoluo Pills | CFDA |
| 226 | Asari Radix et Rhizoma | Shangjin Zhenggu Tincture | CFDA |
| 227 | Asari Radix et Rhizoma | Shaolin Dieda Zhitong Plaster | CFDA |
| 228 | Asari Radix et Rhizoma | Shierwei Chiyinkang Powder | CFDA |
| 229 | Asari Radix et Rhizoma | Shidaoping Powder | CFDA |
| 230 | Asari Radix et Rhizoma | Shuangxin Bidouyan Granules | CFDA |
| 231 | Asari Radix et Rhizoma | Tianma Zhuanggu Pills | CFDA |
| 232 | Asari Radix et Rhizoma | Tongdi Capsules | CFDA |
| 233 | Asari Radix et Rhizoma | Wantong Jingu Tablets | CFDA |
| 234 | Asari Radix et Rhizoma | Weikean Capsules | CFDA |

| | | | |
|---|---|---|---|
| 235 | Asari Radix et Rhizoma | Wumei Pills | CFDA |
| 236 | Asari Radix et Rhizoma | Xiaoke Pingchuan Oral Liquid | CFDA |
| 237 | Asari Radix et Rhizoma | Xinli Zhenggu Spray | CFDA |
| 238 | Asari Radix et Rhizoma | Yaoyong Moxa Stick Moxibustion | CFDA |
| 239 | Asari Radix et Rhizoma | Zushima Fengshi Plaster | CFDA |
| 240 | Asari Radix et Rhizoma | Qufenggutong Cataplasm | CFDA |
| 241 | Asari Radix et Rhizoma | Chanma Zhengtong Tincture | CFDA |
| 242 | Asari Radix et Rhizoma | Chanwu Cataplasm | CFDA |
| 243 | Aristolochiae Mollissimae Herba | Yishen Juanbi Pills | MOH (volume 1) |
| 244 | Aristolochiae Mollissimae Herba | Shangshi Zhentong Plaster | MOH (volume 2) |
| 245 | Aristolochiae Mollissimae Herba | Shaolin Zhenggu Jing | MOH (volume 6) |
| 246 | Aristolochiae Mollissimae Herba | Shaolin Zhenggujing Tincture | MOH (volume 6) |
| 247 | Aristolochiae Mollissimae Herba | Duzhong Zhuanggu Pills[b] | MOH (volume 18) |
| 248 | Aristolochiae Mollissimae Herba | Fufang Quanshen Tablets | MOH (volume 10) |
| 249 | Aristolochiae Mollissimae Herba | Duzhong Zhuanggu Capsules[b] | MOH (volume 12) |
| 250 | Aristolochiae Mollissimae Herba | Fengshining Vinum[b] | MOH (volume 19) |
| 251 | Aristolochiae Mollissimae Herba | Qufeng Chushi Vinum | MOH (volume 13) |
| 252 | Aristolochiae Mollissimae Herba | Shenlong Vinum | MOH (volume 15) |
| 253 | Aristolochiae Mollissimae Herba | Sanshe Vinum | MOH (volume 1) |
| 254 | Aristolochiae Mollissimae Herba | Fufang Fengshi Vinum | CFDA |
| 255 | Aristolochiae Tuberosae Radix | Jinzhu Zhixie Tablets | MOH |
| 256 | Aristolochiae Tuberosae Radix | Zhushalian Capsules | MOH (volume 14) |

[a] Traditional Chinese patent medicine contains both Aristolochiae Fructus and Asari Radix et Rhizoma.

[b] Traditional Chinese patent medicine contains both Aristolochiae Mollissimae Herba and Asari Radix et Rhizoma.

Chinese Pharmacopoeia (Ch.P)

Drug Standard of Ministry of Health People’s Republic of China (MOH)

Drug Standard of China Food and Drug Administration (CFDA)

**Table S2. Species information for Aristolochiaceous and non-Aristolochiaceous plants used in this study.**

| species name (pinyin) | vernacular | Latin name | sample number | voucher No. | GenBank No. ITS2 | GenBank No. *psbA-trnH* | sample from |
|---|---|---|---|---|---|---|---|
| **1.Fangji group** | | | | | | | |
| guang fang ji | Fangji/ Teng fangji | *Aristolochia fangchi** | 2 | YC0663MT01-02 | KM092120-21 | KM213881-82 | Guangxi, Guangzhou |
| yi ye ma dou ling | Hanzhong fangji | *Aristolochia kaempferi* f. *heterophylla** | 4 | YC0678MT01-04 | KM092140-43 | KM213954-56 | Guangxi, Hubei |
| er ye ma dou ling | Heimian fangji | *Aristolochia tagala** | 6 | YC0672MT01-06 | KM092167-72 | KM213875-80 | Yunnan, Hainan |
| fen fang ji | Fangji | *Stephania tetrandra*# | 3 | YC0200MT04-06 | KM092353 | KM233992-94 | Beijing, Hebei |
| mu fang ji | Mu fangji | *Cocculus orbiculatus*# | 3 | YC0486MT01-03 | KM092305-07 | KM233987-89 | Jiangxi |
| zhang ye mu fang ji | Fangji | *Cocculus laurifolius*# | 4 | YC0697MT01-04 | KM092301-04 | KM233990 | Yunnan |
| bian fu ge | Fangji ge | *Menispermum dauricum*# | 6 | YC0146MT38-43 | KM092314-19 | KM233938-42 | Jilin |
| cang bai cheng gou feng | Tu fangji | *Diploclisia glaucescens* | 1 | YC0700MT01 | KM092312 | KM233935 | Guangzhou |
| **2.Mutong group** | | | | | | | |
| guan mu tong | Guan mutong | *Aristolochia manshuriensis** | 5 | YC0469MT01-05 | KM092153-57 | KM213940-43 | Jilin; Korea |
| mu tong | Mutong | *Akebia quinata*# | 11 | YC0408MT01-11 | KM092246-56 | KM233999-4003 | Jiangxi, Guangzhou, Chongqing |
| san ye mu tong | | *Akebia trifoliata*# | 7 | YC0409MT01-07 | KM092257-62,67 | KM234004-08 | Jiangxi, Chongqing, Shanghai |
| bai mu tong | | *Akebia trifoliata* var. *australis*# | 4 | YC0410MT01-03, 05 | KM092263-66 | KM233997-98 | Guangzhou, Wuhan |
| xiao mu tong | Chuan mutong | *Clematis armandii*# | 4 | YC0431MT01-03, 06 | KM092275-78 | KM233932-33 | Chongqing, Yunnan, Guangzhou |
| xiu qiu teng | | *Clematis montana*# | 5 | YC0608MT01-05 | KM092290-94 | KM233927-31 | Sichuan |
| cu chi tie xian lian | Da mutong | *Clematis argentilucida* | 4 | YC0466MT04-07 | KM092271-74 | KM233955,73-75 | Jiangxi, Sichuan |
| dun e tie xian lian | Xi mutong | *Clematis peterae* | 1 | YC0696MT01 | KM092295 | KM233926 | Yunnan |
| da ye tie xian lian | Mutong hua | *Clematis heracleifolia* | 2 | YC0682MT01-02 | KM092283-84 | KM233924-25 | Beijing, shanxi |
| zhu guo tie xian lian | Hua mutong | *Clematis uncinata*# | 1 | YC0803MT01 | KM092308 | / | Guizhou |
| qing feng teng | Tu mutong | *Sinomenium acutum*# | 5 | YC0182MT04, 07-08,12,14 | KM092336-40 | KM233947-48 | Beijing, Henan, Hangzhou |
| wei ling xian | Hei mutong | *Clematis chinensis*# | 4 | YC0630MT01-03,08 | KM092279-82 | KM233984-86 | Jilin, Chongqing |
| mian tuan tie xian lian | | *Clematis hexapetala*# | 5 | YC0485MT01-02, 04-05,07 | KM092285-89 | KM233979-83 | Jilin, Anhui |
| dong bei tie xian lian | | *Clematis manshurica* | 5 | YC0470MT01-03, 06-07 | KM092296-300 | KM233976-78 | Jilin, Anhui |
| tong tuo mu | Tongcao | *Tetrapanax papyrifer* | 4 | YC0243MT02-05 | KM092354-57 | KM233969-72 | Chongqing |
| xi ma shan jing jie hua | | *Stachyurus himalaicus* | 2 | YC0640MT01-02 | KM092351-52 | KM233951 | Yunnan |

| | | | | | | | |
|---|---|---|---|---|---|---|---|
| zhong guo jing jie hua | Xiao tongcao | *Stachyurus chinensis* | 4 | YC0455MT03-06 | KM092347-50 | KM233949-50 | Jiangxi |
| qing jia ye | | *Helwingia japonica* | 3 | YC0444MT01, PS1755MT01-02 | KM092313, GQ434854-55 | KM233991, GQ435474-75 | Chongqing, Sichuan |
| **3.Qingmuxiang group** | | | | | | | |
| bei ma dou ling | Qing muxiang | *Aristolochia contorta*[*] | 4 | YC0111MT11-14 | KM092107-10 | KM213915-18 | Jilin |
| ma dou ling | Qing muxiang | *Aristolochia debilis*[*] | 3 | YC0407MT07-09 | KJ739355-57 | KM213924-26 | Jiangxi |
| bao xing ma dou ling | Qing muxiang | *Aristolochia moupinensis* | 1 | YC0675MT01 | KM092164 | KM213868 | Yunnan |
| yun mu xiang | Qing muxiang | *Aucklandia lappa*[#] | 3 | YC0139MT09, 11,14 | KJ721533,35, 38 | KM233918-20 | Anhui, Hebei, Henan |
| chuan mu xiang | Muxiang | *Vladimiria souliei*[#] | 3 | YC0537MT01, 06-07 | KJ721563-65 | KM233952-54 | Sichuan |
| tu mu xiang | Qing muxiang | *Inula helenium* | 3 | YC0339MT02, 08,12 | KJ721571,77, 81 | KM233936-37 | Henan, Anhui, Tibet |
| qian li xiang | Qing muxiang | *Murraya paniculata* | 6 | YC0054MT02-07 | KM092320-25 | KM233943-46 | Guangxi, Guizhou, Vietnam |
| yun nan qing feng teng | Qing muxiang | *Sabia yunnanensis* | 1 | YC0698MT01 | KM092335 | KM233963 | Yunnan |
| **4.Baimaoteng group** | | | | | | | |
| xun gu feng | Baimaoteng | *Aristolochia mollissima*[*] | 6 | YC0514MT01-06 | KM092158-63 | KM213933-38 | Guangzhou, Henan |
| bai ying | | *Solanum lyratum* | 6 | YC0668MT01-06 | KM092341-46 | KM233964-68 | Hebei, Guangxi |
| **5.Zhushalian group** | | | | | | | |
| zhu sha lian | Zhushalian | *Aristolochia tuberosa*[*] | 4 | YC0681MT01-04 | KM092173-76 | KM213957-59 | Chongqing, Sichuan |
| hong yao zi | | *Pteroxygonum giraldii* | 0 | | EU580725[a], DQ406627[a] | | |
| **6.Shancigu group** | | | | | | | |
| shan ci gu | Shancigu | *Asarum sagittarioides* | 2 | YC0691MT01-02 | KM092220-21 | / | Guangxi |
| du suan lan | Shancigu | *Pleione bulbocodioides* | 4 | YC0555MT01-04 | KM092326-29 | KM233956-58 | Kunming, Guangzhou |
| yun nan du suan lan | Shancigu | *Pleione yunnanensis* | 5 | YC0556MT01-02, 04-06 | KM092330-34 | KM233959-62 | Yunnan |
| du juan lan | Shancigu | *Cremastra appendiculata* | 3 | YC0210MT07-09 | KM092309-11 | KM233934 | Chongqing, Henan, Kunming |
| qing niu dan | Shancigu | *Tinospora sagittata* | 6 | YC0633MT01-02, 05-08 | KM092358-63 | KM233995-96 | Shanghai, Guangzhou, Chongqing |
| **7.Madouling group** | | | | | | | |
| bei ma dou ling | Madouling | *Aristolochia contorta*[*] | 9 | YC0111MT05-10, 15-17 | KM092111-16 | KM213903-10 | Liaoning, Shandong, Guizhou, Yunnan |
| ma dou ling | Madouling | *Aristolochia debilis*[*] | 4 | YC0407MT03-06 | KJ739352-54 | KM213919-22 | Henan, Jiangxi |
| qiao mai ye da bai he | Guang douling | *Cardiocrinum cathayanum* | 3 | YC0487MT01-03 | KM092268-70 | KM233921-23 | Jiangxi |
| ma tong ling | Tu madouling | *Hemsleya graciliflora* | 0 | | JF976558-61[a] | JN044838-41[a] | |
| **8.others** | | | | | | | |
| bei ma dou ling | Tianxianteng | *Aristolochia contorta*[*] | 4 | YC0111MT01-04 | KM092103-06 | KM213911-14 | Jilin |

| | | | | | | | |
|---|---|---|---|---|---|---|---|
| ma dou ling | Tianxianteng | *Aristolochia debilis*[*] | 2 | YC0407MT01-02 | KJ739350-51 | KM213923,27 | Henan, Chongqing |
| guang xi ma dou ling | | *Aristolochia kwangsiensis*[*] | 8 | YC0660MT01-08 | KM092145-52 | KM213883-90 | Guangxi, Guangzhou, Chongqing |
| tong cheng hu | | *Aristolochia fordiana* | 2 | YC0662MT01-02 | KM092123-24 | KM213949-50 | Guangxi |
| bian se ma dou ling | | *Aristolochia versicolor* | 3 | YC0661MT01-03 | KM092177-79 | KM213869-71 | Guangxi |
| nan yue ma dou ling | | *Aristolochia howii* | 3 | YC0664MT01-03 | KM092137-39 | KM213944-46 | Guangzhou |
| mu ben ma dou ling | | *Aristolochia arborea* | 1 | YC0665MT01 | KM092101 | KM213939 | Guangzhou |
| da ye ma dou ling | | *Aristolochia kaempferi*[*] | 1 | YC0666MT01 | KM092144 | KM213874 | Guangzhou |
| mei li ma dou ling | | *Aristolochia elegans*[*] | 3 | YC0667MT01-03 | KM092117-19 | KM213930-32 | Guangzhou, Yunnan |
| da hua ma dou ling | | *Aristolochia grandiflora* | 2 | YC0670MT01-02 | KM092129-30 | KM213872-73 | Guangzhou, Yunnan |
| ju hua ma dou ling | | *Aristolochia gigantea* | 1 | YC0671MT01 | KM092128 | KM213902 | Yunnan |
| ruo jing ma dou ling | | *Aristolochia fimbriata* | 1 | YC0673MT01 | KM092122 | KM213948 | Yunnan |
| yan dou ma dou ling | | *Aristolochia gibertii* | 2 | YC0669MT01-02 | KM092126-27 | KM213952-53 | Guangzhou |
| xi zang ma dou ling | | *Aristolochia griffithii* | 1 | YC0674MT01 | KM092131 | KM213951 | Tibet |
| hai nan ma dou ling | | *Aristolochia hainanensis* | 5 | YC0676MT01-05 | KM092132-36 | KM213891-95 | Hainan |
| huang mao ma dou ling | | *Aristolochia fulvicoma* | 1 | YC0679MT01 | KM092125 | KM213901 | Hainan |
| ou zhou ma dou ling | | *Aristolochia clematitis*[*] | 1 | YC0680MT01 | KM092102 | KM213947 | Kew Botanical Garden |
| hai nan xian guo dou ling | | *Thottea hainanensis* | 5 | YC0677MT01-05 | KM092242-45 | KM213896-900 | Hainan |
| bei xi xin | | *Asarum heterotropoides* var. *mandshuricum*[#] | 6 | YC0365MT01-06 | KM092198-203 | / | Jilin, Liaoning |
| han cheng xi xin | | *Asarum sieboldii* var. *seoulense* | 6 | YC0478MT01-06 | KM092229-34 | / | Jilin |
| hua xi xin | xixin | *Asarum sieboldii*[#] | 7 | YC0607MT01-07 | KM092222-28 | / | Shanxi, Anhui |
| xiao ye ma ti xiang | | *Asarum ichangense* | 5 | YC0504MT01-05 | KM092208-12 | / | Jiangxi, Guangzhou, Anhui |
| du heng | | *Asarum forbesii*[#] | 6 | YC0471MT01-06 | KM092187-92 | / | Jiangxi, Anhui |
| di hua xi xin | | *Asarum geophilum* | 5 | YC0625MT01-05 | KM092193-97 | / | Guangxi, Guangzhou |
| qing cheng xi xin | | *Asarum splendens*[*] | 4 | YC0623MT01-04 | KM092237-40 | / | Guangzhou, Shanxi |
| wei hua xi xin | | *Asarum caudigerum* | 2 | YC0624MT01-02 | KM092183-84 | / | Guangzhou |
| jin er huan | | *Asarum insigne* | 1 | YC0690MT01 | KM092213 | / | Guangxi |
| chang jing jin er huan | | *Asarum longerhizomatosum* | 3 | YC0685MT01-03 | KM092214-16 | / | Guangzhou, Guangxi |
| da ye ma ti xiang | | *Asarum maximum* | 1 | YC0686MT01 | KM092217 | / | Hubei |
| dan ye xi xin | | *Asarum himalaicum*[*] | 4 | YC0688MT01-04 | KM092204-07 | / | Chongqing, Sichuan, Gansu |
| shuang ye xi xin | | *Asarum caulescens* | 1 | YC0687MT01 | KM092185 | / | Chongqing |
| duan wei xi xin | | *Asarum caudigerellum* | 1 | YC0694MT01 | KM092182 | / | Sichuan |
| jiu gong shan xi xin | | *Asarum campaniflorum* | 2 | YC0695MT01-02 | KM092180-81 | / | Hubei |
| chang mao xi xin | | *Asarum pulchellum* | 2 | YC0693MT01-02 | KM092218-19 | / | Chongqing, Guizhou |
| zhou hua xi xin | | *Asarum crispulatum* | 1 | YC0692MT01 | KM092186 | / | Sichuan |
| ma ti xiang | | *Saruma henryi* | 1 | YC0684MT01 | KM092241 | / | Shanxi |

| | | | | | | | |
|---|---|---|---|---|---|---|---|
| | | *Aristolochia sp.* | 2 | YC0800MT01-02 | KM092165-66 | KM213928-29 | Guangxi |
| | | *Asarum sp.* | 2 | YC0801MT01-02 | KM092235-36 | / | Guangxi |
| deng long xi xin | | *Asarum inflatum* | 0 | | FJ428622,28[a] | | |
| ba shan xi xin | | *Asarum bashanense* | 0 | | FJ428619-20[a] | | |
| hua ye xi xin | | *Asarum cardiophyllum* | 0 | | FJ428651[a] | | |
| tong qian xi xin | | *Asarum debile* | 0 | | FJ428644[a] | | |
| qi yang xi xin | | *Asarum magnificum* | 0 | | FJ428640[a] | | |
| wu ling xi xin | | *Asarum wulingense* | 0 | | FJ428636[a] | | |
| zi bei xi xin | | *Asarum porphyronotum* | 0 | | FJ428627[a] | | |
| chuan dian xi xin | | *Asarum delavayi* | 0 | | FJ428626[a] | | |
| chuan bei xi xin | | *Asarum chinense* | 0 | | FJ428623[a] | | |
| nan chuan xi xin | | *Asarum nanchuanense* | 0 | | FJ428618[a] | | |
| yun nan xi xin | | *Asarum yunnanense* | 0 | | FJ428656[a] | | |
| xiang gang xi xin | | *Asarum hongkongense* | 0 | | FJ428635[a] | | |
| cheng kou xi xin | | *Asarum chengkouense* | 0 | | FJ428616[a] | | |

[*] FDA: Botanicals known or suspected to contain Aristolochic acid

[#] FDA: Botanicals which may be adulterated with Aristolochic acid

[a] download from GenBank

/ failed to obtain

**Table S3. List of universal primers and reaction conditions for ITS2 and *psbA-trnH*.**

| Marker | Name of primers | Primer sequences 5′-3′ | PCR reaction conditions |
|---|---|---|---|
| **ITS2** | ITS2F | ATGCGATACTTGGTGTGAAT | 94℃ 5 min |
| | ITS3R | GACGCTTCTCCAGACTACAAT | 94℃ 30 s, 56℃ 30 s, 72℃ 45 s, 40 cycles |
| | | | 72℃ 10 min |
| ***psbA-trnH*** | fwd PA | GTTATGCATGAACGTAATGCTC | 95℃ 4 min |
| | rev TH | CGCGCATGGTGGATTCACAATCC | 94℃ 30 s, 55℃ 1 min, 72℃ 1 min, 35 cycles |
| | | | 72℃ 10 min |

**Table S4. List of the 11 groups for realtime PCR in combination with Taqman probe based on ITS2 sequence similarity from Aristolochiaceae.**

| Family | Genus | Group | Latin name | Haplotype | voucher No. |
|---|---|---|---|---|---|
| **Aristolochiaceae** | *Aristolochia* | M1 | *Aristolochia moupinensis* | A | YC0675MT01 |
| | | M1 | *Aristolochia versicolor* | A | YC0661MT01 |
| | | M1 | | B | YC0661MT02-03 |
| | | M1 | *Aristolochia kaempferi* | A | YC0666MT01 |
| | | M1 | *Aristolochia manshuriensis* | A | YC0469MT01-05 |
| | | M1 | *Aristolochia fangchi* | A | YC0663MT01-02 |
| | | M1 | *Aristolochia kwangsiensis* | A | YC0660MT01-05,07-08 |
| | | M1 | | B | YC0660MT06 |
| | | M1 | *Aristolochia howii* | A | YC0664MT01-03 |
| | | M1 | *Aristolochia fulvicoma* | A | YC0679MT01 |
| | | M1 | *Aristolochia mollissima* | A | YC0514MT01-06 |
| | | M1 | *Aristolochia arborea* | A | YC0665MT01 |
| | | M1 | *Aristolochia griffithii* | A | YC0674MT01 |
| | | M1 | *Aristolochia kaempferi* f. *heterophylla* | A | YC0678MT01-02,04 |
| | | M1 | | B | YC0678MT03 |
| | | M1 | *Aristolochia hainanensis* | A | YC0676MT01-03,05 |
| | | M1 | | B | YC0676MT04 |
| | | M1 | *Aristolochia sp.* | A | YC0800MT01 |
| | | M1 | | B | YC0800MT02 |
| | | M2 | *Aristolochia grandiflora* | A | YC0670MT01-02 |
| | | M2 | *Aristolochia elegans* | A | YC0667MT01-03 |
| | | M2 | *Aristolochia fimbriata* | A | YC0673MT01 |
| | | M2 | *Aristolochia gibertii* | A | YC0669MT01-02 |
| | | M2 | *Aristolochia gigantea* | A | YC0671MT01 |
| | | M3 | *Aristolochia contorta* | A | YC0111MT01,04-05,08,11,14 |
| | | M3 | | B | YC0111MT02,09-10,12-13 |
| | | M3 | | C | YC0111MT06 |
| | | M3 | | D | YC0111MT03 |
| | | M3 | | E | YC0111MT07 |
| | | M4 | *Aristolochia clematitis* | A | YC0680MT01 |
| | | M4 | *Aristolochia tuberosa* | A | YC0681MT01-04 |
| | | M5 | *Aristolochia fordiana* | A | YC0662MT02 |
| | | M5 | | B | YC0662MT01 |
| | | M6 | *Aristolochia debilis* | A | YC0407MT01 |
| | | M6 | | B | YC0407MT02 |

| | | | | | |
|---|---|---|---|---|---|
| | | M6 | | C | YC0407MT04-08 |
| | | M6 | | D | YC0407MT09 |
| | | M7 | *Aristolochia tagala* | A | YC0672MT02 |
| | | M7 | | B | YC0672MT01 |
| | | M7 | | C | YC0672MT03 |
| | | M7 | | D | YC0672MT04-06 |
| | *Asarum* | X1 | *Asarum caudigerum* | A | YC0624MT01-02 |
| | | X1 | *Asarum sieboldii* | A | YC0607MT01-06 |
| | | X1 | | B | YC0607MT07 |
| | | X1 | *Asarum sieboldii* var. *seoulense* | A | YC0478MT01,03 |
| | | X1 | | B | YC0478MT02,04 |
| | | X1 | | C | YC0478MT05-06 |
| | | X1 | *Asarum geophilum* | A | YC0625MT03,05 |
| | | X1 | | B | YC0625MT04 |
| | | X1 | | C | YC0625MT01-02 |
| | | X1 | *Asarum heterotropoides* var. *mandshuricum* | A | YC0365MT02-03 |
| | | X1 | | B | YC0365MT06 |
| | | X1 | | C | YC0365MT04 |
| | | X1 | | D | YC0365MT01,05 |
| | | X1 | *Asarum ichangense* | A | YC0504MT01-05 |
| | | X2 | *Asarum crispulatum* | A | YC0692MT01 |
| | | X2 | *Asarum longerhizomatosum* | A | YC0685MT02 |
| | | X2 | | B | YC0685MT01 |
| | | X2 | | C | YC0685MT03 |
| | | X2 | *Asarum sagittarioides* | A | YC0691MT01-02 |
| | | X2 | *Asarum splendens* | A | YC0623MT01-04 |
| | | X2 | *Asarum campaniflorum* | A | YC0695MT01-02 |
| | | X2 | *Asarum sp.* | A | YC0801MT01-02 |
| | | X2 | *Asarum insigne* | A | YC0690MT01 |
| | | X2 | *Asarum forbesii* | A | YC0471MT01-03 |
| | | X2 | | B | YC0471MT04 |
| | | X2 | | C | YC0471MT05 |
| | | X2 | | D | YC0471MT06 |
| | | X2 | *Asarum maximum* | A | YC0686MT01 |
| | | X3&MT | *Asarum caudigerellum* | A | YC0694MT01 |
| | | X3&MT | *Asarum pulchellum* | A | YC0693MT01-02 |
| | | X3&MT | *Asarum himalaicum* | A | YC0688MT01-04 |

| | | | | | |
|---|---|---|---|---|---|
| | | X3&MT | *Asarum caulescens* | A | YC0687MT01 |
| | *Saruma* | X3&MT | *Saruma henryi* | A | YC0684MT01 |
| | *Thottea* | Hn | *Thottea hainanensis* | A | YC0677MT01-02,04-05 |

**Table S5. Primers and hydrolysis probes used in AAs-containing plant detection**

| oligonucleotides | sequence (5′-3′) | amplicon (bp) |
|---|---|---|
| Hn-L | CCAAACGACGGCCCTAATG | 84 |
| Hn-R | CACGGCCGAAAAGACTTACC | |
| Hn-P | FAM-CACGGCGAGTGGTGGCAGCGT-TAMARA | |
| M1-L | GTTGGCTGAAASSCTWGGC | 136 |
| M1-R | AGGCRRACGGTTAGSGTC | |
| M1-P | FAM-CACGACAAGTGGTGGCTCGGCC-TAMARA | |
| M2-L | GGCGAGGTTGGCTGAAAA | 205 |
| M2-R | GCTTAAACTCAGCGGGTGT | |
| M2-P | FAM-CTGCGTGGCTCGAAGTCGTGTCC-TAMARA | |
| M3-L | AAAATCCAGGCCCCTCGG | 196 |
| M3-R | CTTAAACTCAGCGGGTGGTC | |
| M3-P | FAM-CTCCRAGCTCCCCGGCCTCT-TAMARA | |
| M4-L | CTCGAAGTCGTGCCYGYGA | 204 |
| M4-R | GCTTTTCCYRGTTCGCTCSC | |
| M4-P | FAM-CTTGGGAGGCKGCGAGGAMC-TAMARA | |
| M5-L | TTGCACCCGAGGCCACTA | 216 |
| M5-R | GAARCGACGGAGGGCRAAG | |
| M5-P | FAM-ACAGTTGGTGGCTCGAAGCTCCCC-TAMARA | |
| M6-L | AAAATCCAGGCCCCTCGG | 76 |
| M6-R | ACTTCGAGCCTGGCAAGAG | |
| M6-P | FAM-TCGCGGCGCGACAACTGGTG-TAMARA | |
| M7-L | CCCCMRGGTGAAAAGGCAAG | 123 |
| M7-R | TCCTCGCAGCCTTCCCAG | |
| M7-P | FAM-ACGGCGCGACTACTGGTGGC-TAMARA | |
| X1-L | TATAGGRCGCGGAKATTGGC | 93 |
| X1-R | ACCACCACTAGACGTATCRC | |
| X1-P | FAM-TCCGTTCAAATCMTTGYGCGGTTTGC-TAMARA | |
| X2-L | CCRCATCCATCTCGGATAYAGGA | 150 |
| X2-R | TCGACACGYCCTYCTGTCAA | |
| X2-P | FAM-ATTGGCTAYCCGTTCRAATCCTTGCG-TAMARA | |
| X3&MT-L | CTGCTTGGGCGTCATGCTA | 134 |
| X3&MT-R | GTATCGYAGTCCRCCAAAGG | |
| X3&MT-P | FAM-TCGCTCCCACATCCATCTCAATAGAAGTG-TAMARA | |

**Table S6. Detection of AA Ⅰ and Ⅱ for all species using UHPLC-HR-MS analysis.**

| Latin name | name | voucher No. | AA Ⅰ | AA Ⅱ |
|---|---|---|---|---|
| *Aristolochia contorta* | a1-1* | YC0111MT07-08,15 | + | + |
| *Aristolochia contorta* | a1-2* | YC0111MT01-03 | + | - |
| *Aristolochia contorta* | a1-3* | YC0111MT11-13 | + | + |
| *Aristolochia fangchi* | a2 | YC0663MT01-02 | + | + |
| *Aristolochia manshuriensis* | a3 | YC0469MT01-03 | + | + |
| *Aristolochia mollissima* | a4 | YC0514MT02,04-05 | + | - |
| *Aristolochia kaempferi* f. *heterophylla* | a5 | YC0678MT01-02 | + | - |
| *Aristolochia howii* | a6 | YC0664MT01-03 | + | + |
| *Aristolochia kaempferi* | a7 | YC0666MT01 | + | + |
| *Aristolochia moupinensis* | a8 | YC0675MT01 | + | + |
| *Aristolochia tuberosa* | a9 | YC0681MT01-02 | + | + |
| *Aristolochia hainanensis* | a10 | YC0676MT01,05 | + | - |
| *Aristolochia versicolor* | a11 | YC0661MT01,03 | + | - |
| *Aristolochia fordiana* | a12 | YC0662MT01-02 | + | + |
| *Aristolochia griffithii* | a13 | YC0674MT01 | + | + |
| *Aristolochia fulvicoma* | a14 | YC0679MT01 | + | - |
| *Aristolochia sp.* | a15 | YC0800MT01-02 | + | - |
| *Aristolochia debilis* | a33-1* | YC0407MT04-06 | + | + |
| *Aristolochia debilis* | a33-2* | YC0407MT07-09 | + | - |
| *Aristolochia debilis* | a33-3* | YC0407MT01-02 | + | + |
| *Aristolochia arborea* | a34 | YC0665MT01 | + | - |
| *Aristolochia fimbriata* | a35 | YC0673MT01 | + | - |
| *Aristolochia gibertii* | a36 | YC0669MT01-02 | + | + |
| *Aristolochia gigantea* | a37 | YC0671MT01 | + | - |
| *Aristolochia grandiflora* | a38 | YC0670MT01 | + | - |
| *Aristolochia tagala* | a39 | YC0672MT03-04 | + | - |
| *Asarum heterotropoides* var. *mandshuricum* | a16 | YC0365MT02,06 | + | - |
| *Asarum sieboldii* var. *seoulense* | a17 | YC0478MT01,03,04 | + | - |
| *Asarum sieboldii* | a18 | YC0607MT01,03,05-07 | + | - |
| *Asarum sagittarioides* | a19 | YC0691MT01-02 | + | - |
| *Asarum ichangense* | a20 | YC0504MT01, 04-05 | + | - |
| *Asarum longerhizomatosum* | a21 | YC0685MT01 | + | - |
| *Asarum maximum* | a22 | YC0686MT01 | + | - |
| *Asarum himalaicum* | a23 | YC0688MT01-03 | + | - |
| *Asarum splendens* | a24 | YC0623MT02-04 | + | + |
| *Asarum forbesii* | a25 | YC0471MT01,05 | + | - |
| *Asarum geophilum* | a26 | YC0625MT02-03 | + | - |
| *Asarum insigne* | a27 | YC0690MT01 | + | - |

| | | | | |
|---|---|---|---|---|
| *Asarum crispulatum* | a28 | YC0692MT01 | + | − |
| *Asarum caudigerellum* | a29 | YC0694MT01 | + | − |
| *Asarum sp.* | a30 | YC0801MT01-02 | + | + |
| *Saruma henryi* | a31 | YC0684MT01 | + | − |
| *Thottea hainanensis* | a32 | YC0677MT01-03 | + | + |
| *Aristolochia kwangsiensis* | a40 | YC0660MT01-02,04-05,08 | − | − |
| *Aristolochia elegans* | a41 | YC0667MT01-03 | − | − |
| *Asarum caudigerum* | a42 | YC0624MT01-02 | − | − |
| *Asarum caulescens* | a43 | YC0687MT01 | − | − |
| *Asarum campaniflorum* | a44 | YC0695MT01-02 | − | − |
| *Asarum pulchellum* | a45 | YC0693MT01 | − | − |
| *Stephania tetrandra* | f1 | YC0200MT05-06 | − | − |
| *Cocculus orbiculatus* | f2 | YC0486MT01-03 | − | − |
| *Cocculus laurifolius* | f3 | YC0697MT01-03 | − | − |
| *Menispermum dauricum* | f4 | YC0146MT38-39,43 | − | − |
| *Akebia quinata* | f5 | YC0408MT01-03 | − | − |
| *Akebia trifoliata* | f6 | YC0409MT02-03,07 | − | − |
| *Clematis armandii* | f7 | YC0431MT02-03 | − | − |
| *Clematis argentilucida* | f8 | YC0466MT04-06 | − | − |
| *Clematis peterae* | f9 | YC0696MT01 | − | − |
| *Sabia yunnanensis* | f10 | YC0698MT01 | − | − |
| *Cardiocrinum cathayanum* | f11 | YC0487MT01-03 | − | − |
| *Diploclisia glaucescens* | f12 | YC0700MT01 | − | − |
| *Akebia trifoliata* var. *australis* | f13 | YC0410MT03,05 | − | − |
| *Clematis montana* | f14 | YC0608MT01,04 | − | − |
| *Sinomenium acutum* | f15 | YC0182MT07-08 | − | − |
| *Clematis chinensis* | f16 | YC0630MT03,09 | − | − |
| *Clematis hexapetala* | f17 | YC0485MT04-05 | − | − |
| *Clematis manshurica* | f18 | YC0470MT03 | − | − |
| *Tetrapanax papyrifer* | f19 | YC0243MT02,04 | − | − |
| *Stachyurus chinensis* | f20 | YC0455MT03-04 | − | − |
| *Helwingia japonica* | f21 | YC0444MT01 | − | − |
| *Aucklandia lappa* | f22 | YC0139MT09,11 | − | − |
| *Vladimiria souliei* | f23 | YC0537MT01,06-07 | − | − |
| *Inula helenium* | f24 | YC0339MT02,08 | − | − |
| *Murraya paniculata* | f25 | YC0054MT06-07 | − | − |
| *Solanum lyratum* | f26 | YC0668MT01-02 | − | − |
| *Pleione bulbocodioides* | f27 | YC0555MT01,03-04 | − | − |
| *Pleione yunnanensis* | f28 | YC0556MT12 | − | − |
| *Cremastra appendiculata* | f29 | YC0210MT07-08 | − | − |

| | | | | |
|---|---|---|---|---|
| *Tinospora sagittata* | f30 | YC0633MT06-07 | − | − |

* a1-1: the fruits of *Ar. contorta*; a1-2: the herbs of *Ar. contorta*;

a1-3: the roots and rhizomes of *Ar. contorta*

a33-1: the fruits of *Ar. debilis*; a33-2: the herbs of *Ar. debilis*;

a33-3: the roots and rhizomes of *Ar. debilis*

\+ contain AA

− AA free

**Table S7 Summary of commercial samples identified by DNA Barcoding, Real-Time PCR and UHPLC-HR-MS.**

| Sample number | Vernacular | Species in Chinese Pharmacopoeia | Sample from | Species identified by DNA barcoding | | Rapid detection by Real-Time PCR | Detection by UHPLC-HR-MS | | Mislabelled |
|---|---|---|---|---|---|---|---|---|---|
| | | | | ITS2 identification (Similarity) | *psbA-trnH* identification (Similarity) | | AA Ⅰ | AA Ⅱ | |
| MDL01 | Fangji | *Stephania tetrandra* | Guangzhou, China | *Stephania tetrandra* (100%) | NS | NPS | － | － | NO |
| MDL02 | Fangji | *Stephania tetrandra* | Hebei, China | *Stephania tetrandra* (100%) | NS | NPS | － | － | NO |
| MDL03 | Fangji | *Stephania tetrandra* | Hebei#, China | *Aristolochia fangchi* (100%) | NS | PS | ＋ | － | YES |
| MDL04 | Fangji | *Stephania tetrandra* | Anhui#, China | *Stephania tetrandra* (100%) | *Stephania tetrandra* (100%) | NPS | － | － | NO |
| MDL05 | Zhushalian | *Aristolochia tuberosa* | Anhui, China | *Aristolochia tuberosa* (100%) | *Aristolochia tuberosa* (100%) | PS | ＋ | ＋ | NO |
| MDL06 | Zhushalian | *Aristolochia tuberosa* | Hebei, China | NS | *Dioscorea sp.* (100%) | NPS | － | － | YES |
| MDL07 | Zhushalian | *Aristolochia tuberosa* | Hebei#, China | *Aristolochia tuberosa* (100%) | NS | PS | ＋ | ＋ | NO |
| MDL08 | Xungufeng | *Aristolochia mollissima* | Hebei, China | *Aristolochia mollissima* (100%) | *Aristolochia mollissima* (100%) | PS | ＋ | ＋ | NO |
| MDL09 | Xungufeng | *Aristolochia mollissima* | Anhui, China | *Aristolochia mollissima* (100%) | *Aristolochia mollissima* (100%) | PS | ＋ | ＋ | NO |
| MDL10 | Baiying | *Solanum lyratum* | Hebei, China | *Solanum lyratum* (100%) | *Solanum lyratum* (100%) | NPS | － | － | NO |
| MDL11 | Baiying | *Solanum lyratum* | Anhui, China | *Solanum lyratum* (96%) | *Solanum lyratum* (100%) | NPS | － | － | NO |
| MDL12 | Mutong | *Akebia quinata*, *Akebia trifoliate* and *Akebia trifoliate* var. *australis* | Hebei, China | *Clematis sp.* (99%) | *Clematis sp.* (100%) | NPS | － | － | YES |
| MDL13 | Mutong | *Akebia quinata*, *Akebia trifoliate* and *Akebia trifoliate* var. *australis* | Anhui, China | *Clematis sp.* (98%) | *Clematis sp.*(97%) | NPS | － | － | YES |
| MDL14 | Chuanmutong | *Clematis armandii* and *Clematis montana* | Beijing, China | *Clematis sp.* (99%) | *Clematis sp.* (100%) | NPS | － | － | NO |
| MDL15 | Chuanmutong | *Clematis armandii* and *Clematis montana* | Beijing, China | *Clematis sp.* (100%) | *Clematis sp.* (100%) | NPS | － | － | NO |
| MDL16 | Chuanmutong | *Clematis armandii* and *Clematis montana* | Guangzhou, China | *Clematis sp.* (99%) | NS | NPS | － | － | NO |
| MDL17 | Xixin | *Asarum heterotropoides* var. *mandshuricum*, *Asarum sieboldii* var. | Guangzhou, China | *Asarum sp.* (100%) | NS | PS | ＋ | ＋ | NO |

| | | | | | | | | | |
|---|---|---|---|---|---|---|---|---|---|
| | | *seoulense* and *Asarum sieboldii* | | | | | | | |
| MDL18 | Xixin | *Asarum heterotropoides* var. *mandshuricum*, *Asarum sieboldii* var. *seoulense* and *Asarum sieboldii* | Beijing, China | *Asarum sp.* (100%) | NS | PS | + | − | NO |
| MDL19 | Xixin | *Asarum heterotropoides* var. *mandshuricum*, *Asarum sieboldii* var. *seoulense* and *Asarum sieboldii* | Beijing, China | *Asarum sp.* (100%) | NS | PS | − | − | NO |
| MDL20 | Xixin | *Asarum heterotropoides* var. *mandshuricum*, *Asarum sieboldii* var. *seoulense* and *Asarum sieboldii* | Hebei, China | *Asarum sp.* (97%) | NS | PS | ND | | NO |

No Sequence (NS), Not Detected (ND)

No Positive Signal (NPS): from non-Aristolochiaceous samples

Positive Signal (PS): from Aristolochiaceous samples

# sample from online stores

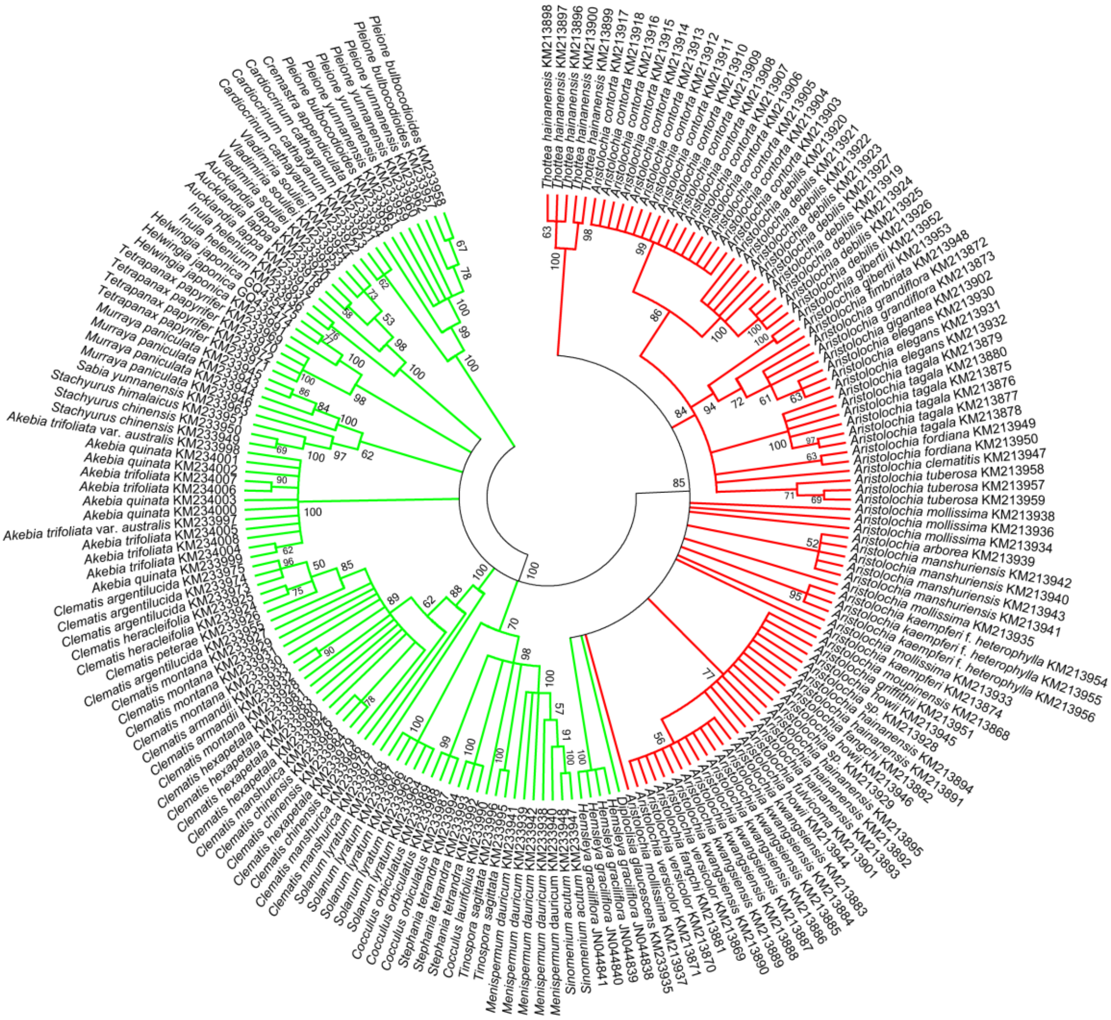

**Fig. S1. The NJ tree constructed from the *psbA-trnH* region from Aristolochiaceae and non-Aristolochiaceae substitutes. The bootstrap scores (1000 replicates) are shown (≥50%) for each branch.**

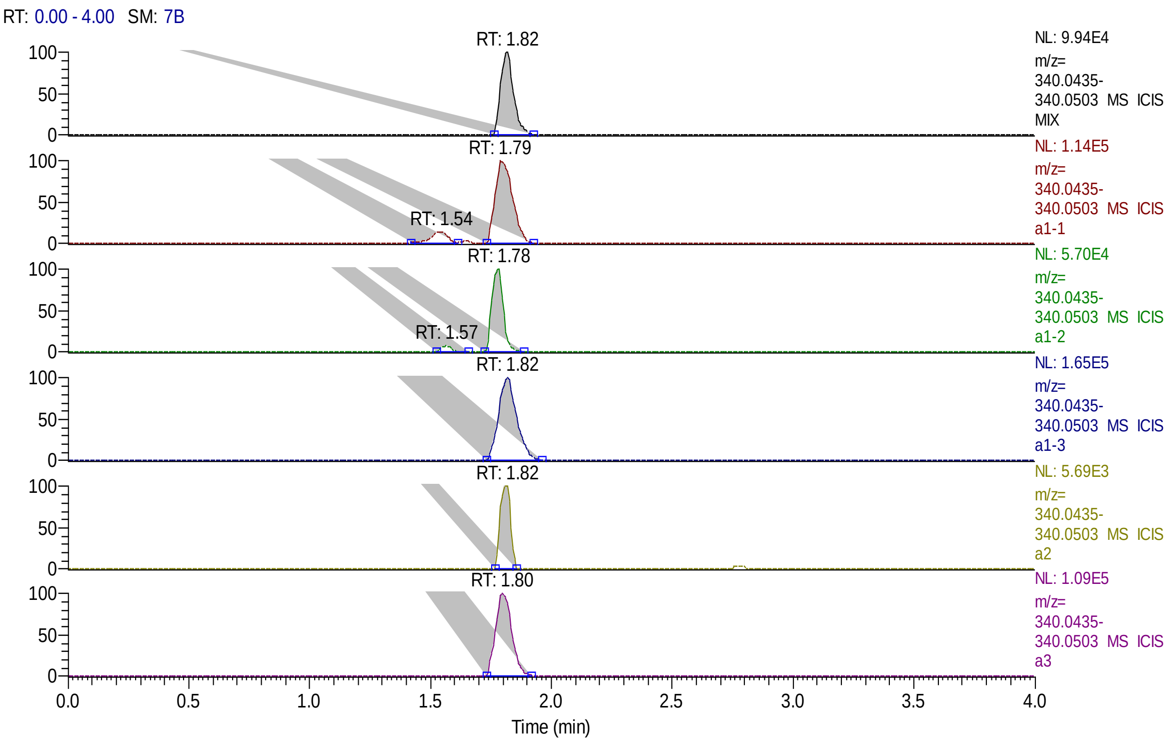


MIX: standard AA Ⅰ; a1-1: the fruits of *Ar. contorta*; a1-2: the herbs of *Ar. contorta*; a1-3: the roots and rhizomes of *Ar. contorta*; a2: *Ar. fangchi*; a3: *Ar. manshuriensis*

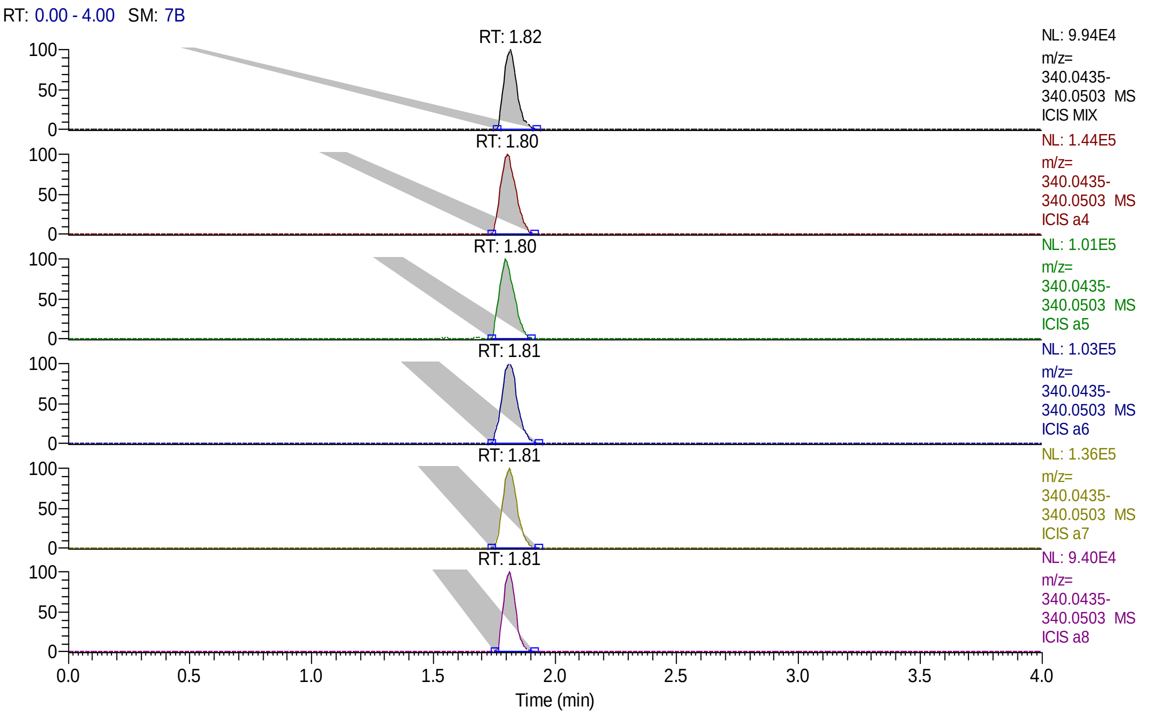


MIX: standard AA Ⅰ; a4: *Ar. mollissima*; a5: *Ar. kaempferi* f. *heterophylla*; a6: *Ar. howii*; a7: *Ar. kaempferi*; a8: *Ar. moupinensis*

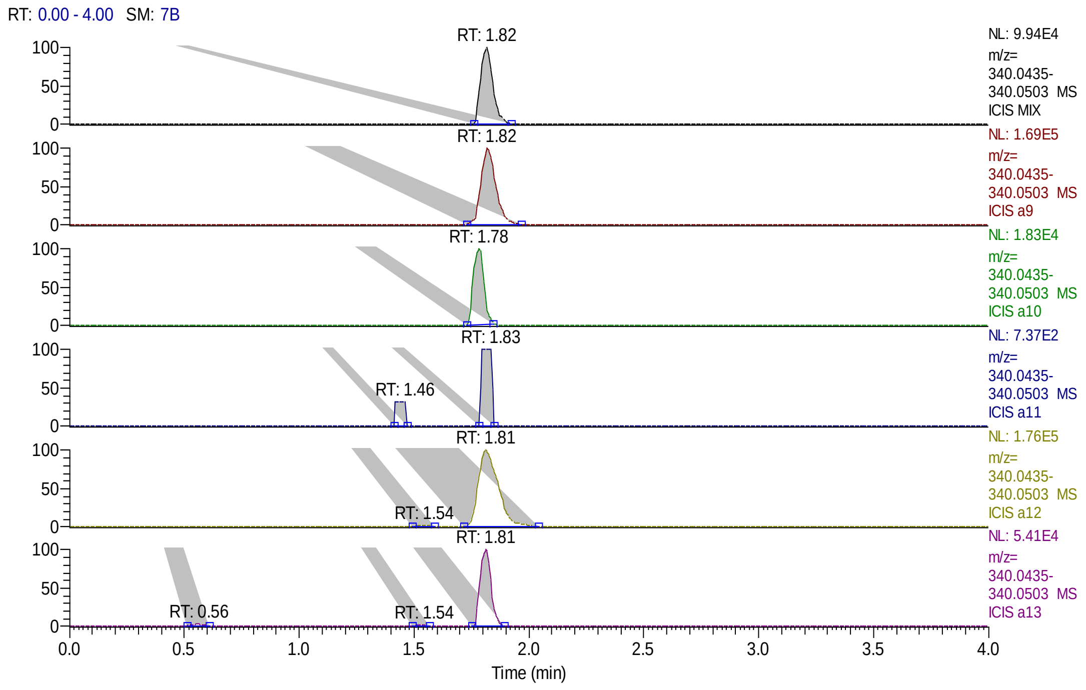


MIX: standard AA Ⅰ; a9: *Ar. tuberosa*; a10: *Ar. hainanensis*;
a11: *Ar. versicolor*; a12: *Ar. fordiana*; a13: *Ar. griffithii*

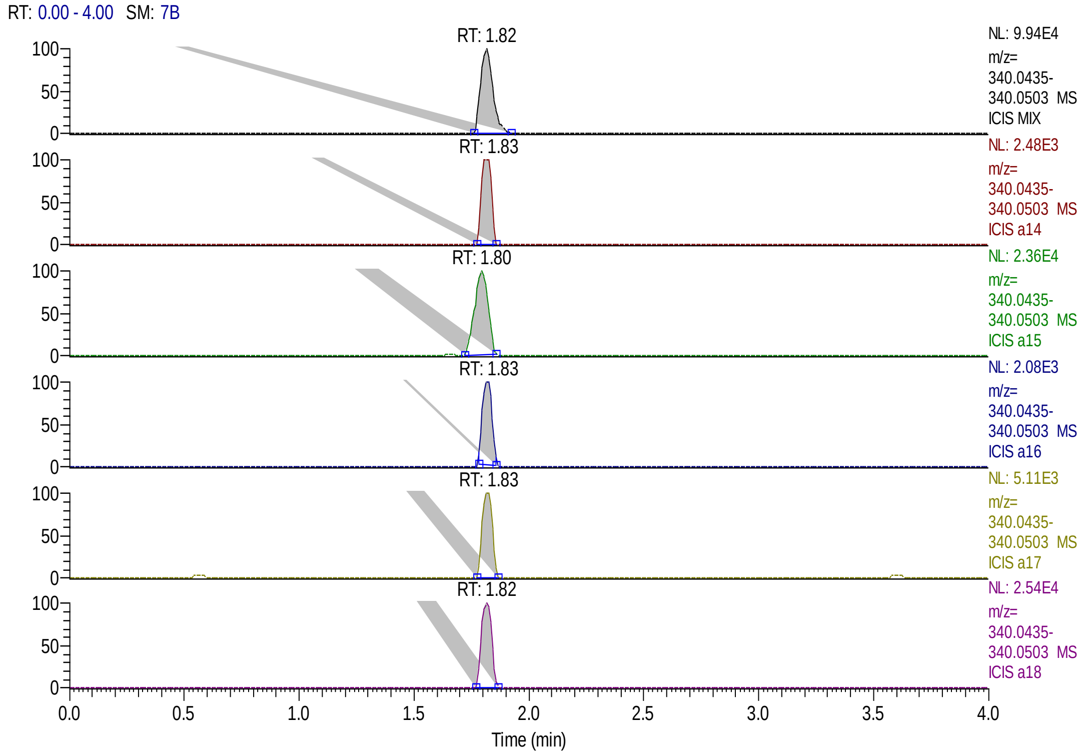


MIX: standard AA Ⅰ; a14: *Ar. fulvicoma*; a15: *Ar. sp.*;
a16: *As. heterotropoides* var. *mandshuricum*;
a17: *As. sieboldii* var. *seoulense*; a18: *As. sieboldii*

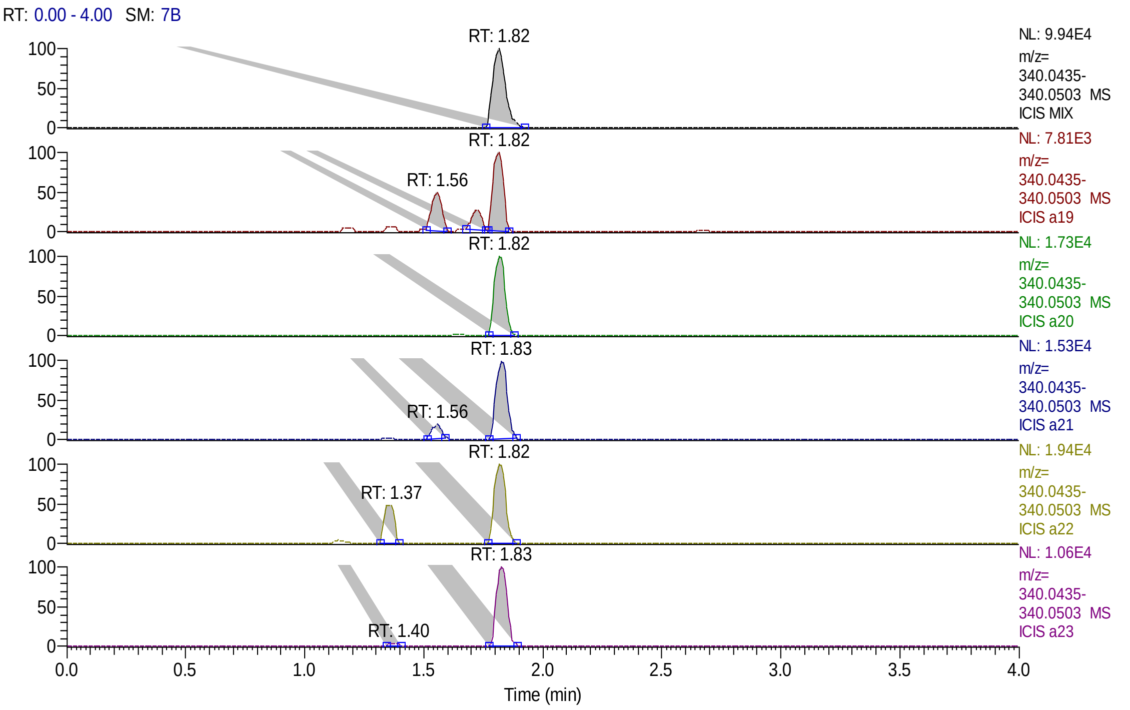


MIX: standard AA Ⅰ; a19: *As. sagittarioides*; a20: *As. ichangense*;
a21: *As. longerhizomatosum*; a22: *As. maximum*; a23: *As. himalaicum*

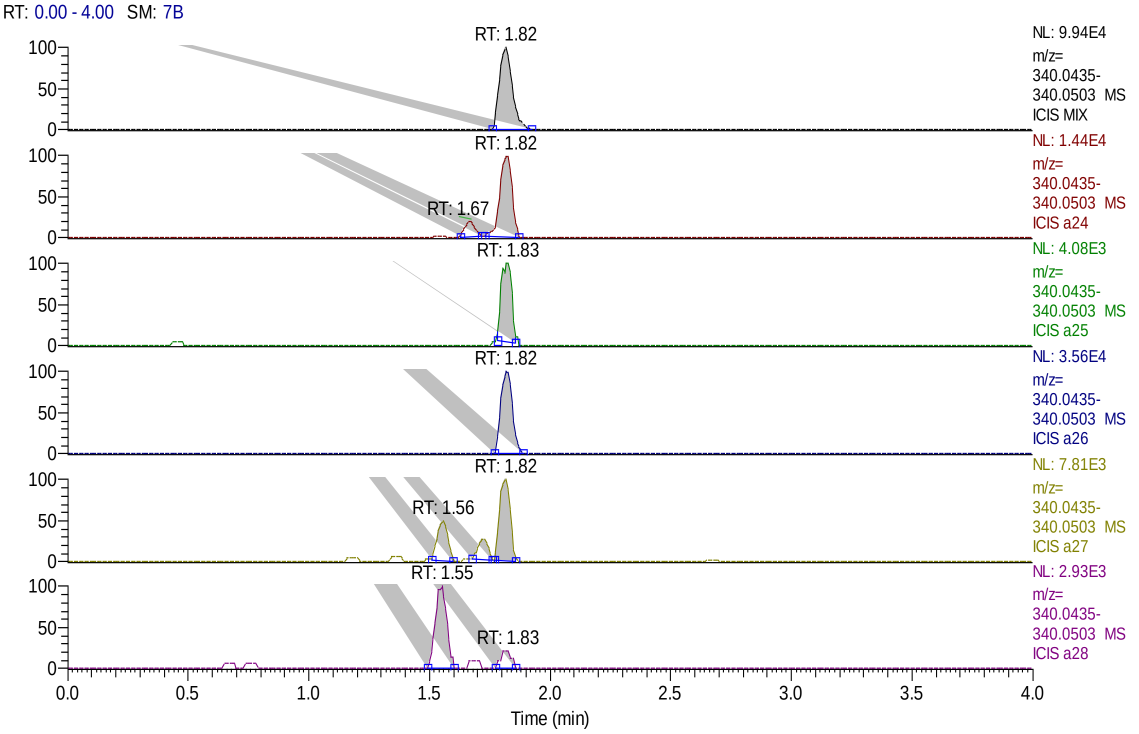


MIX: standard AA Ⅰ; a24: *As. splendens*; a25: *As. forbesii*;
a26: *As. geophilum*; a27: *As. insigne*; a28: *As. crispulatum*

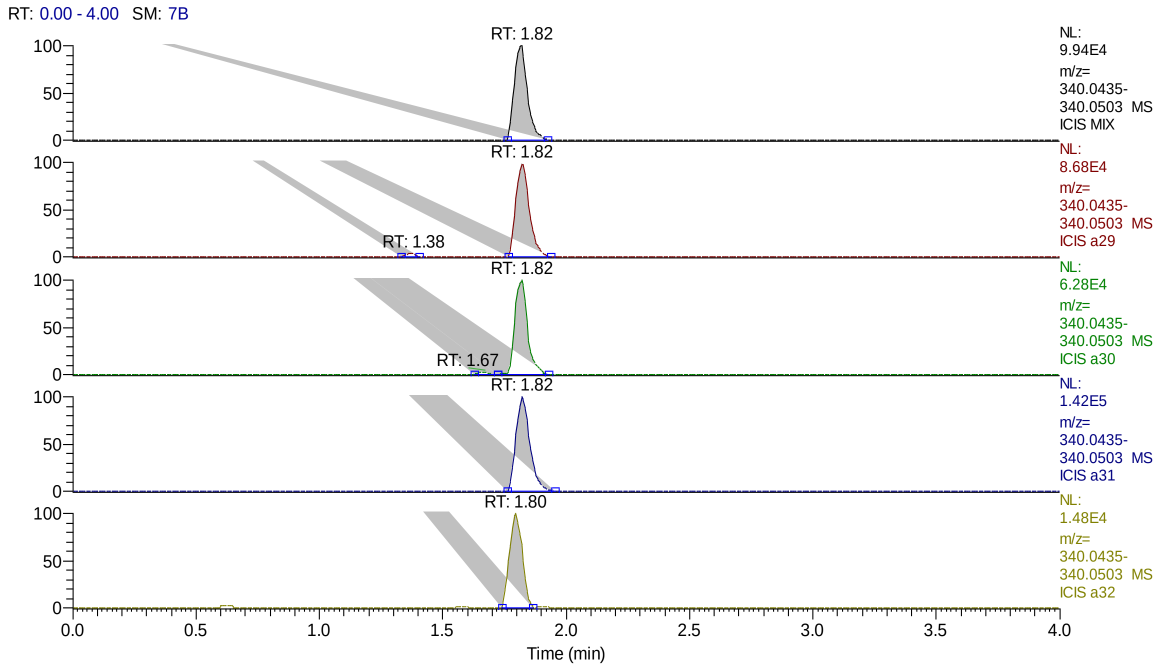


MIX: standard AA; a29: *As. caudigerellum*; a30: *As. sp.*; a31: *Saruma henryi*; a32: *Thottea hainanensis*

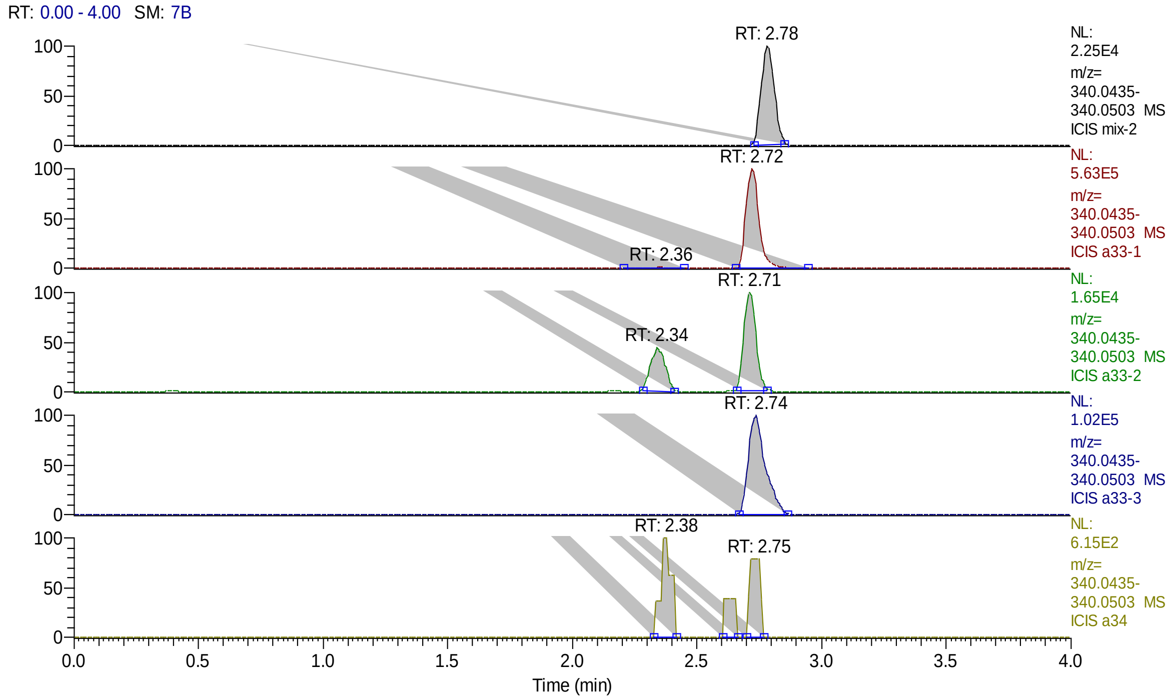


mix-2: standard AA; a33-1: the fruits of *Ar. debilis*; a33-2: the herbs of *Ar. debilis*; a33-3: the roots and rhizomes of *Ar. debilis*; a34: *Ar. arborea*

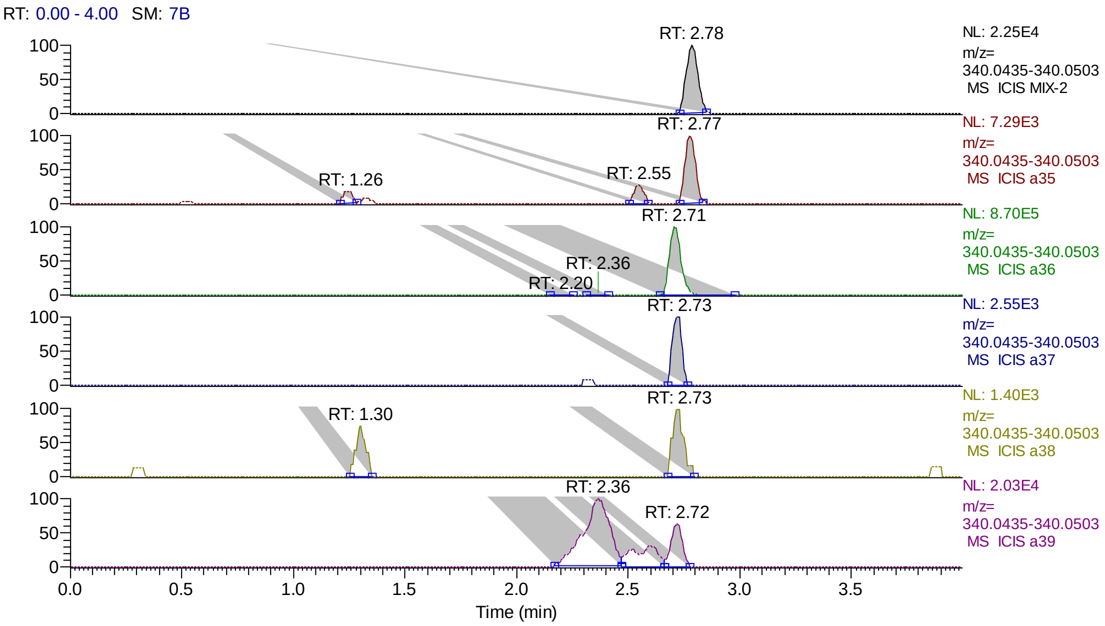


mix-2: standard AA Ⅰ; a35: *Ar. fimbriata*; a36: *Ar. gibertii*; a37: *Ar. gigantea*; a38: *Ar. grandiflora*; a39: *Ar. tagala*

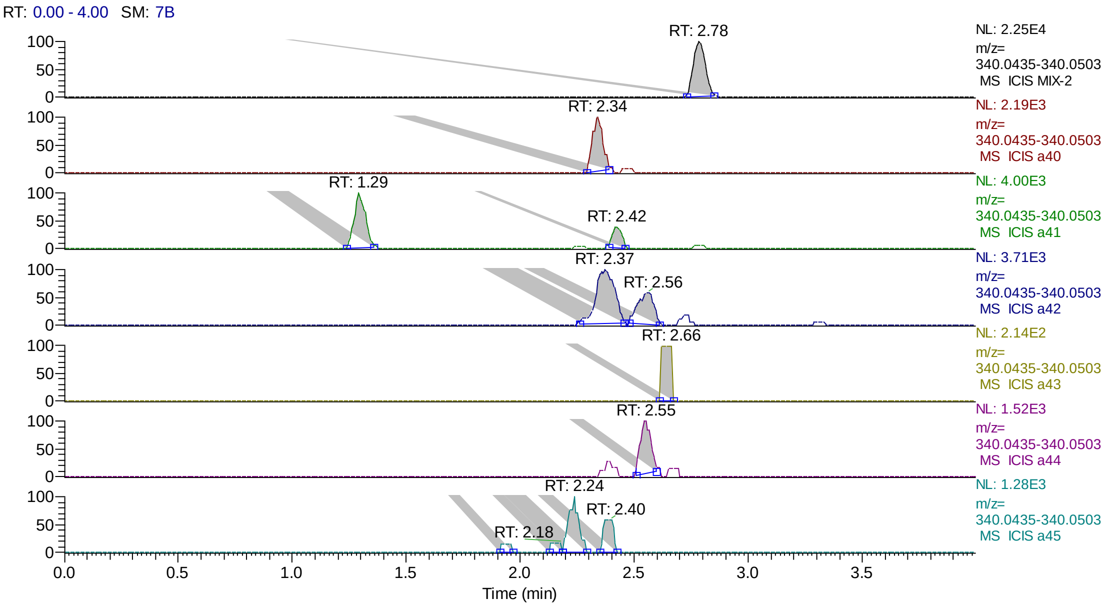


mix-2: standard AA Ⅰ; a40: *Ar. kwangsiensis*; a41: *Ar. elegans*; a42: *As. caudigerum*; a43: *As. caulescens*; a44: *As. campaniflorum*; a45: *As. pulchellum*;

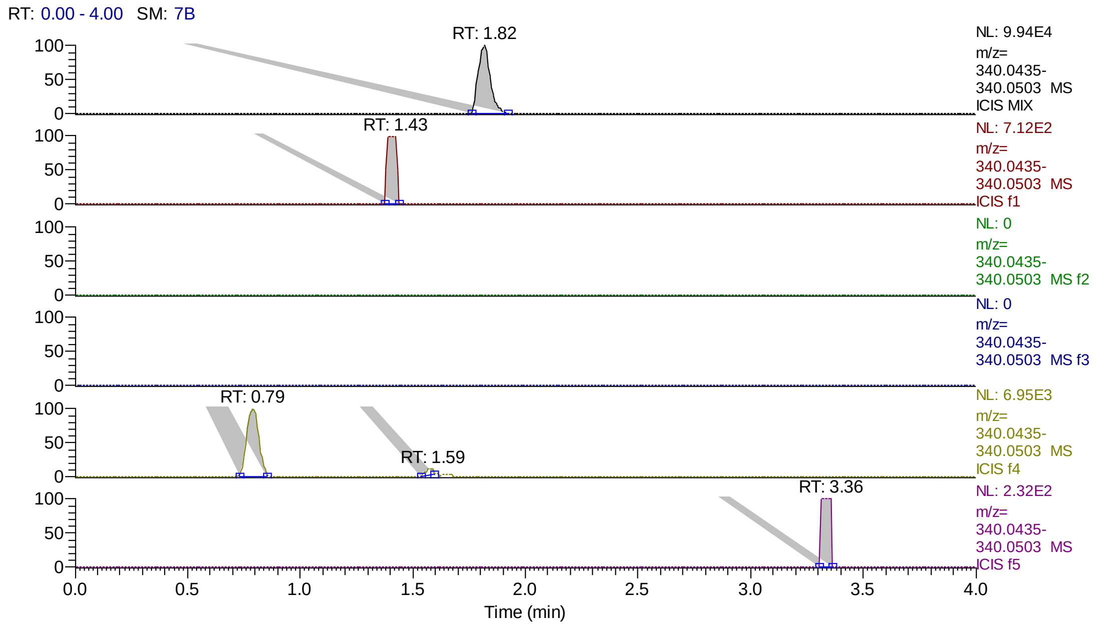


MIX: standard AAⅠ; f1: *Stephania tetrandra*; f2: *Cocculus orbiculatus*;
f3: *Cocculus laurifolius*; f4: *Menispermum dauricum*; f5: *Akebia quinata* ;

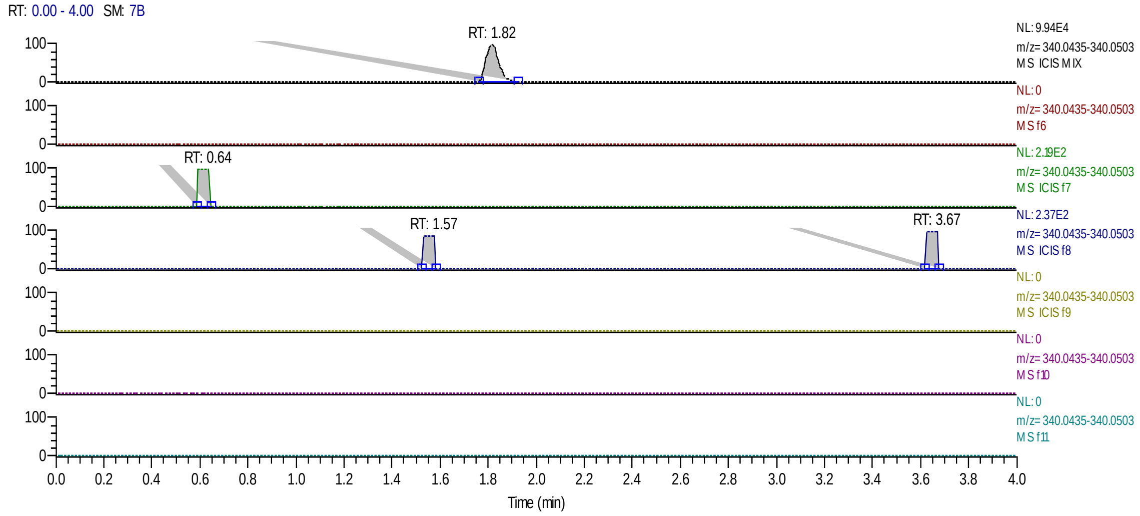


MIX: standard AAⅠ; f6: *Akebia trifoliata*; f7: *Clematis armandii*;
f8: *Clematis argentilucida*; f9: *Clematis peterae*; f10: *Sabia yunnanensis*;
f11: *Cardiocrinum cathayanum*

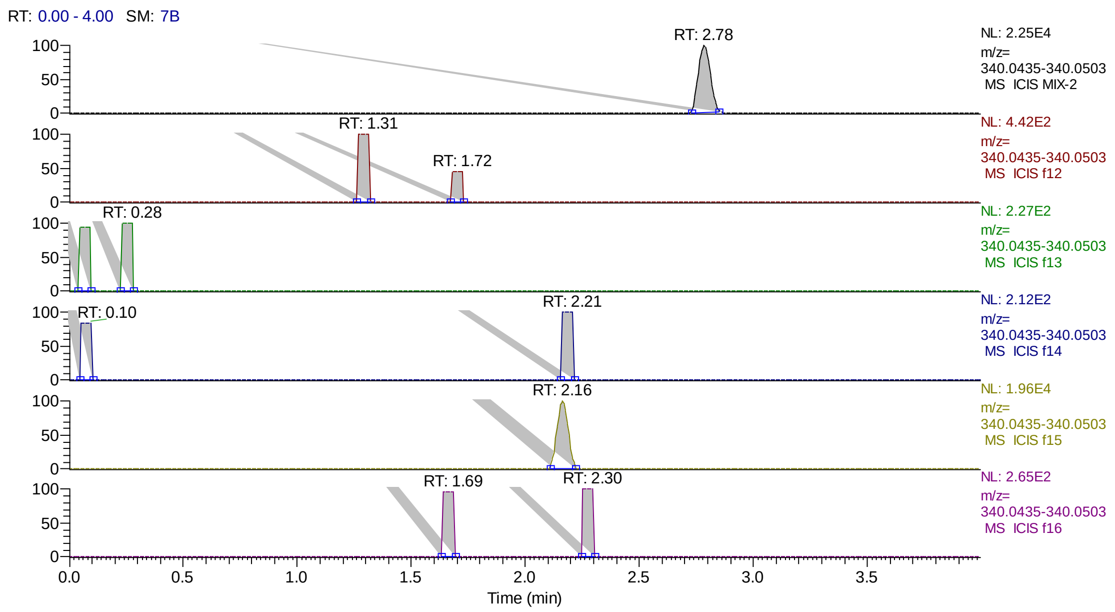


mix-2: standard AAⅠ; f12: *Diploclisia glaucescens*; f13: *Akebia trifoliata* var. *australis*; f14: *Clematis montana*; f15: *Sinomenium acutum*; f16: *Clematis chinensis*;

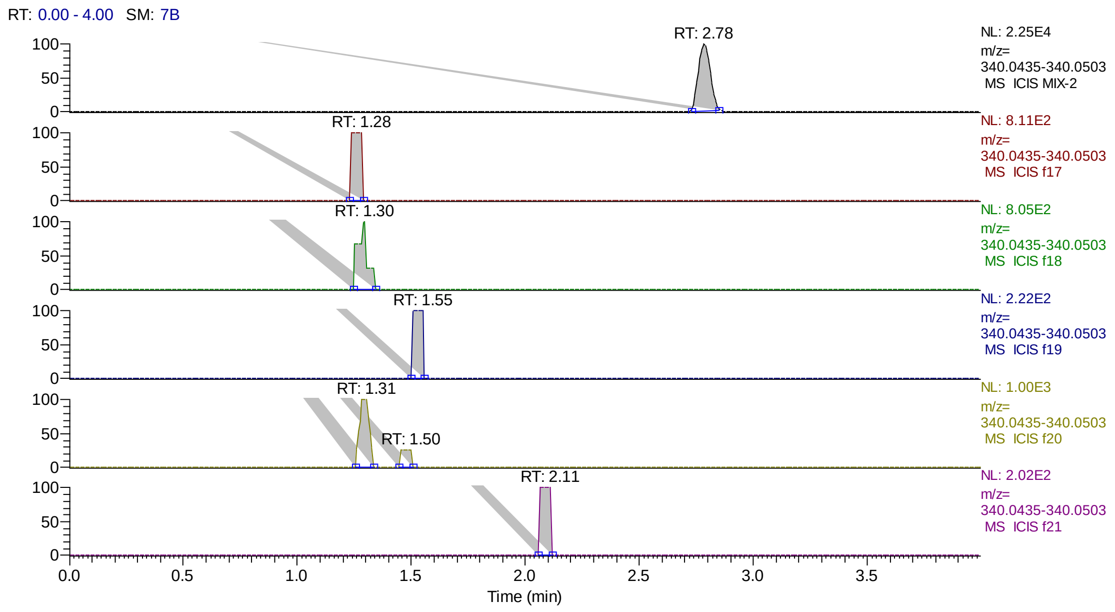


mix-2: standard AAⅠ; f17: *Clematis hexapetala*; f18: *Clematis manshurica*; f19: *Tetrapanax papyrifer*; f20: *Stachyurus chinensis*; f21: *Helwingia japonica*;

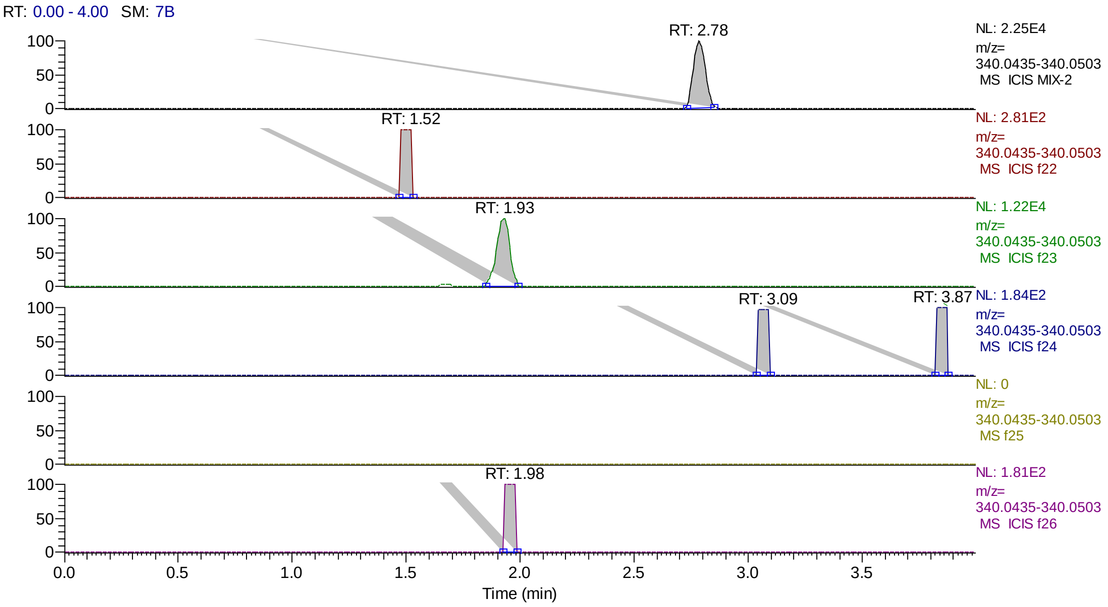


mix-2: standard AA Ⅰ; f22: *Aucklandia lappa*; f23: *Vladimiria souliei*;
f24: *Inula helenium*; f25: *Murraya paniculata*; f26: *Solanum lyratum*;

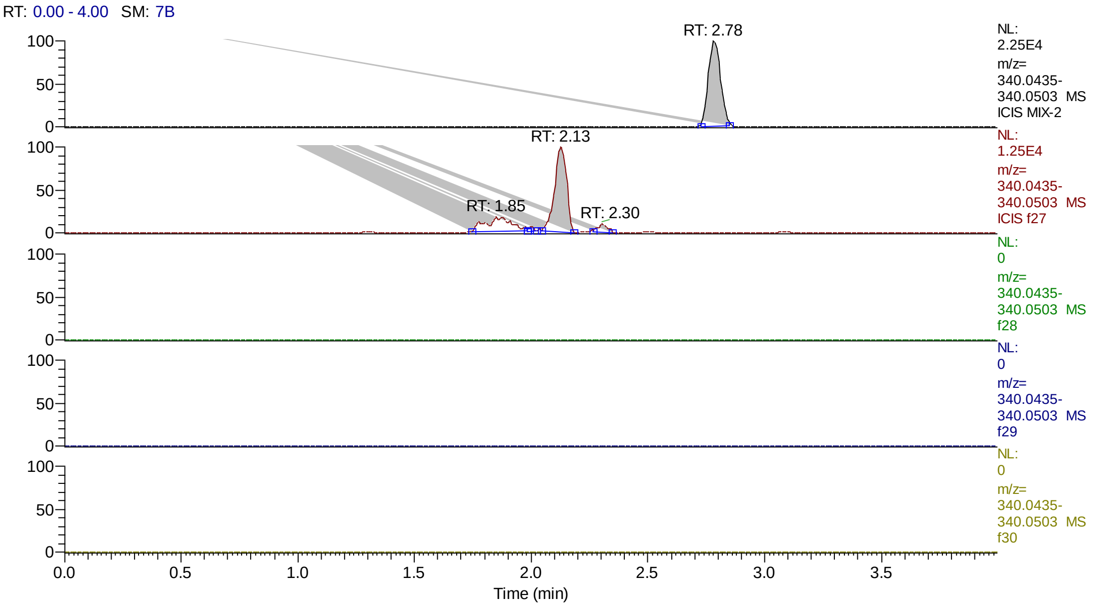


mix-2: standard AA Ⅰ; f27: *Pleione bulbocodioides*; f28: *Pleione yunnanensis*;
f29: *Cremastra appendiculata*; f30: *Tinospora sagittata*;

**Fig. S2. MS profiles of AA Ⅰ for all species in UHPLC-HR-MS analysis. AA Ⅰ was detected at *m/z* 340 0469.**

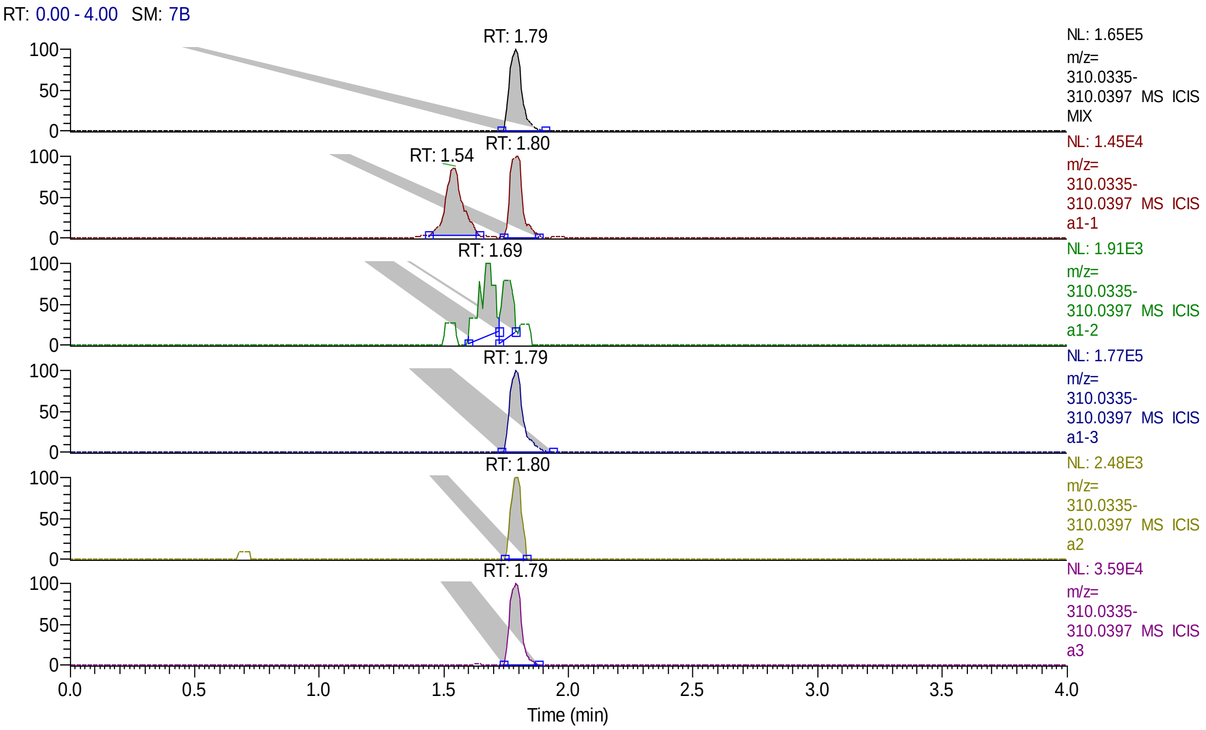


MIX: standard AA II; a1-1: the fruits of *Ar. contorta*; a1-2: the herbs of *Ar. contorta*; a1-3: the roots and rhizomes of *Ar. contorta*; a2: *Ar. fangchi*; a3: *Ar. manshuriensis*

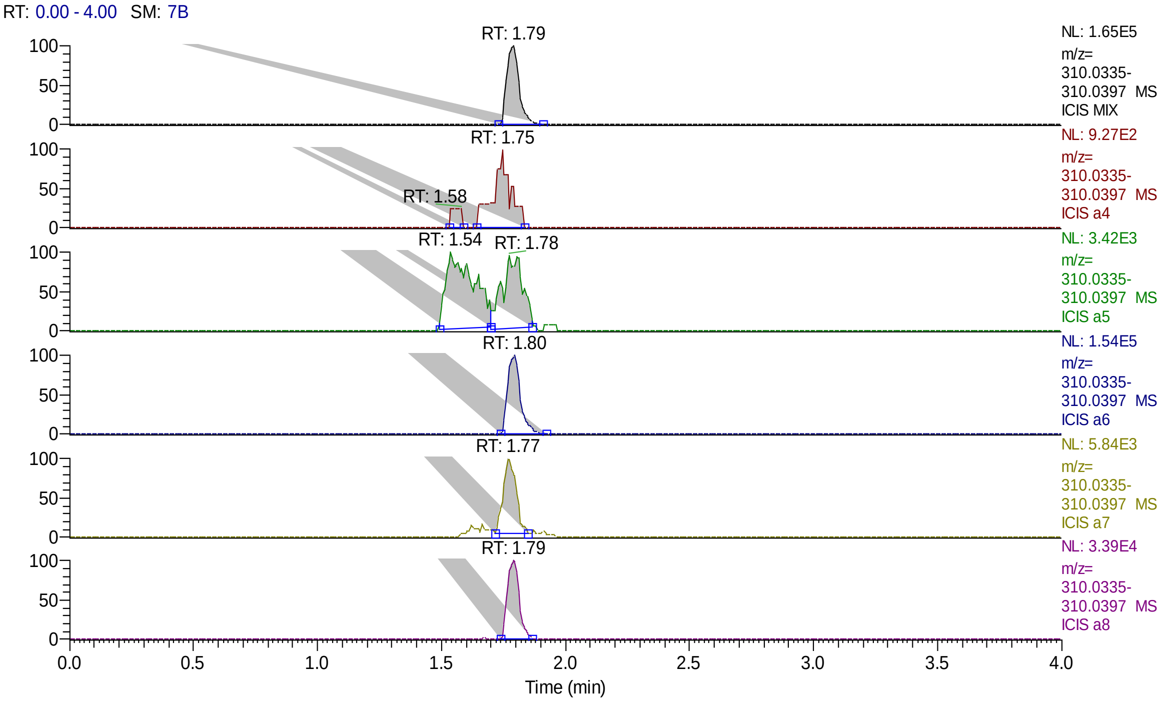


MIX: standard AA II; a4: *Ar. mollissima*; a5: *Ar. kaempferi* f. *heterophylla*; a6: *Ar. howii*; a7: *Ar. kaempferi*; a8: *Ar. moupinensis*

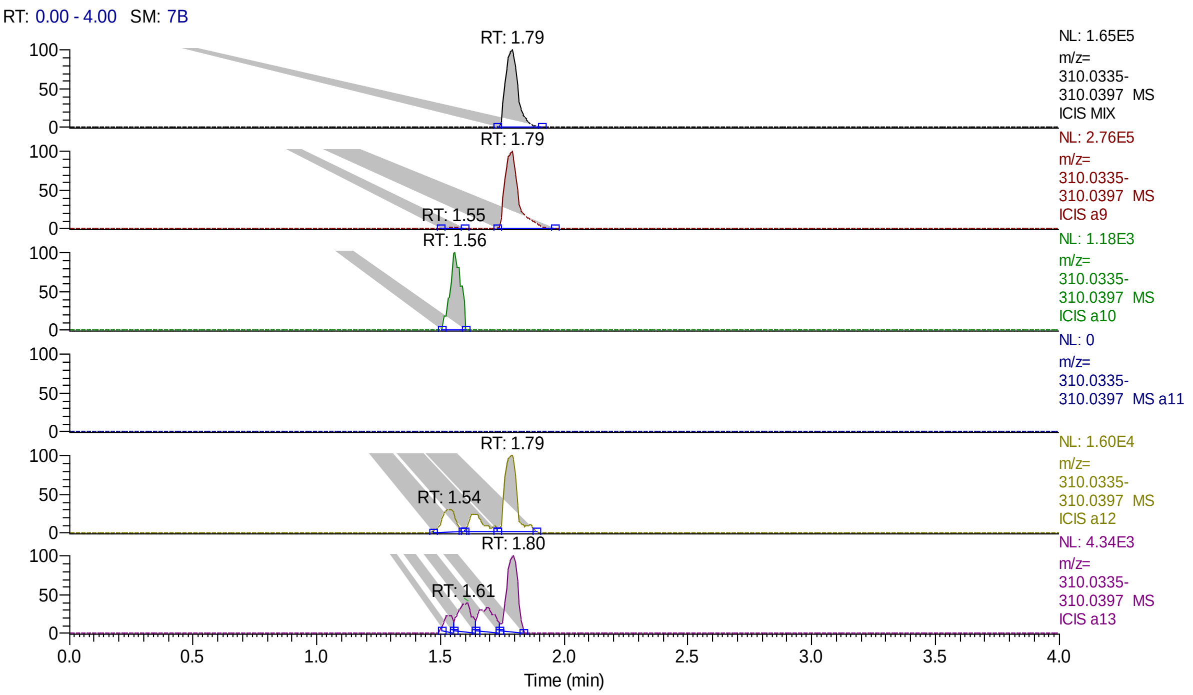


MIX: standard AA Ⅱ; a9: *Ar. tuberosa*; a10: *Ar. hainanensis*;
a11: *Ar. versicolor*; a12: *Ar. fordiana*; a13: *Ar. griffithii*

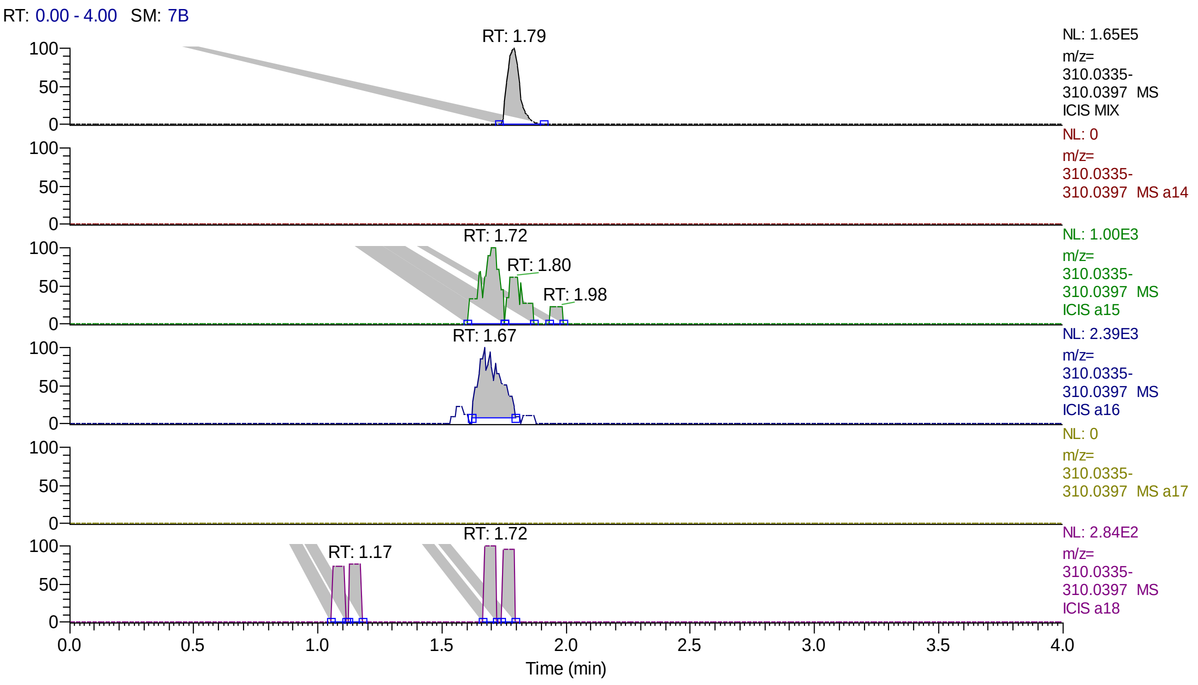


MIX: standard AA Ⅱ; a14: *Ar. fulvicoma*; a15: *Ar. sp.*;
a16: *As. heterotropoides* var. *mandshuricum*;
a17: *As. sieboldii* var. *seoulense*; a18: *As. sieboldii*

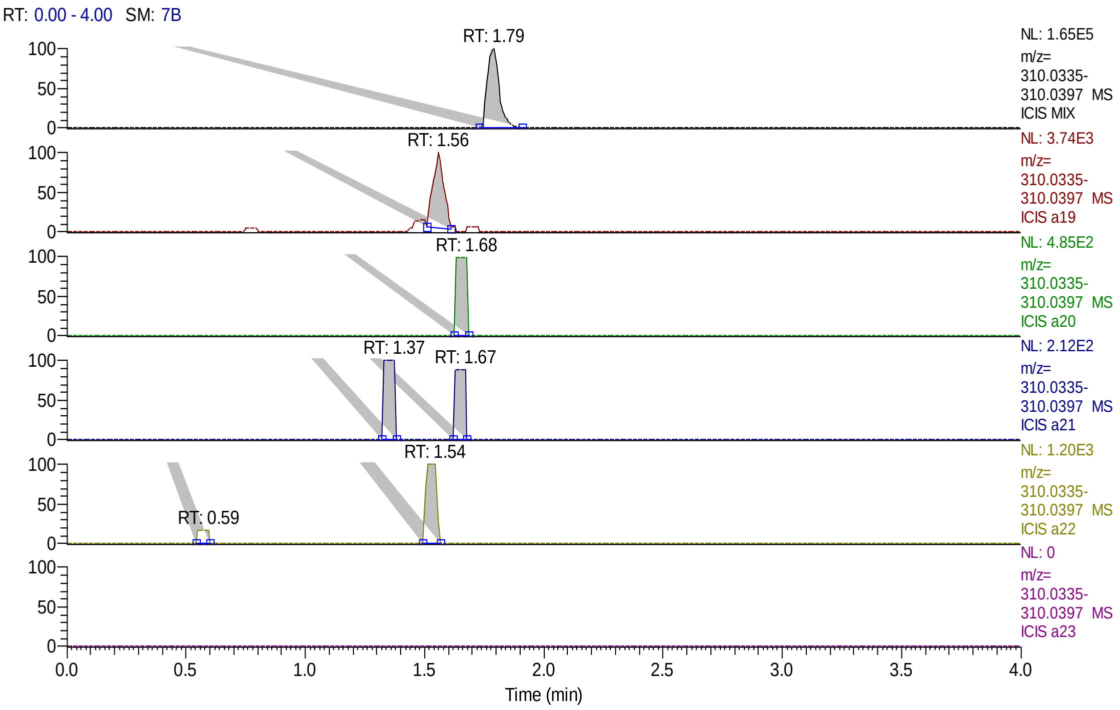


MIX: standard AA Ⅱ; a19: *As. sagittarioides*; a20: *As. ichangense*;
a21: *As. longerhizomatosum*; a22: *As. maximum*; a23: *As. himalaicum*

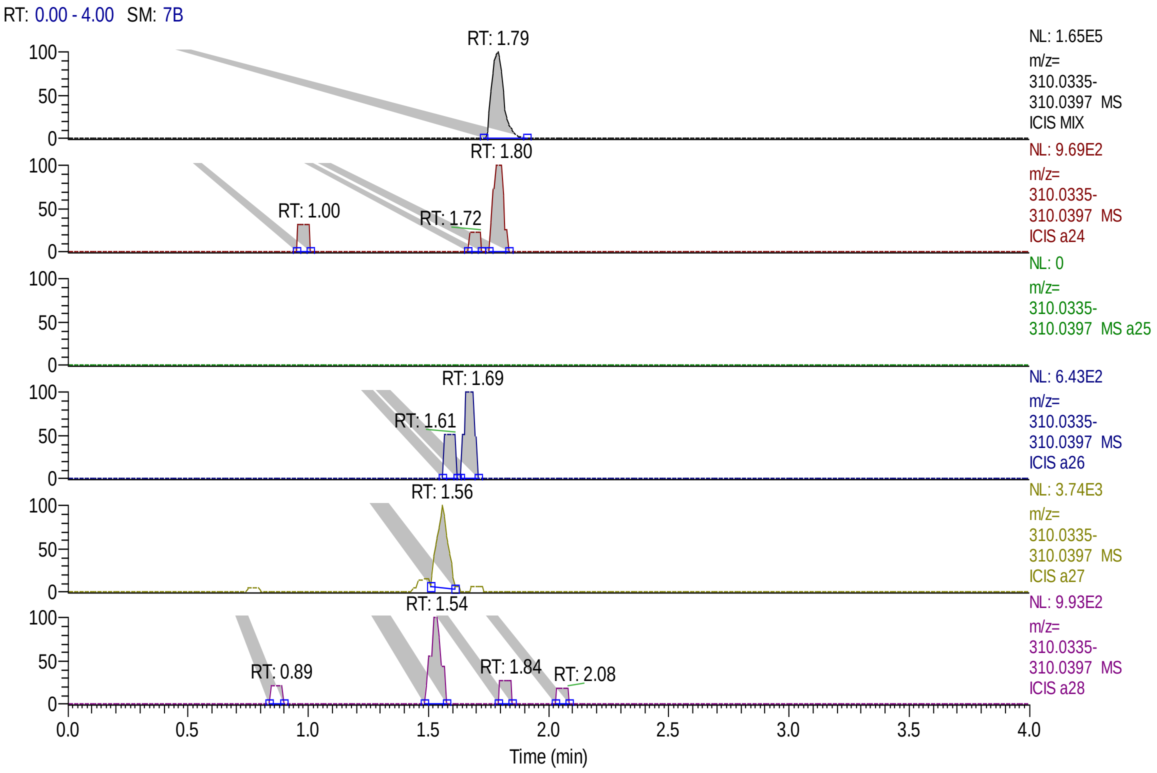


MIX: standard AA Ⅱ; a24: *As. splendens*; a25: *As. forbesii*;
a26: *As. geophilum*; a27: *As. insigne*; a28: *As. crispulatum*;

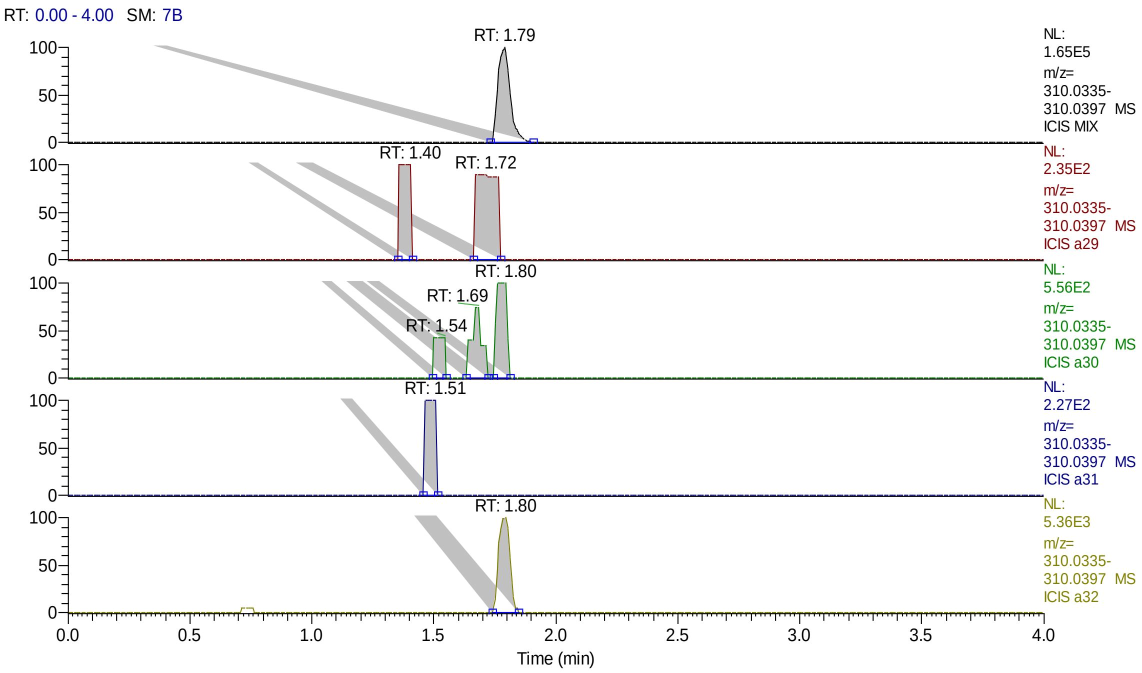


MIX: standard AA II; a29: *As. caudigerellum*; a30: *As. sp.*; a31: *Saruma henryi*; a32: *Thottea hainanensis*

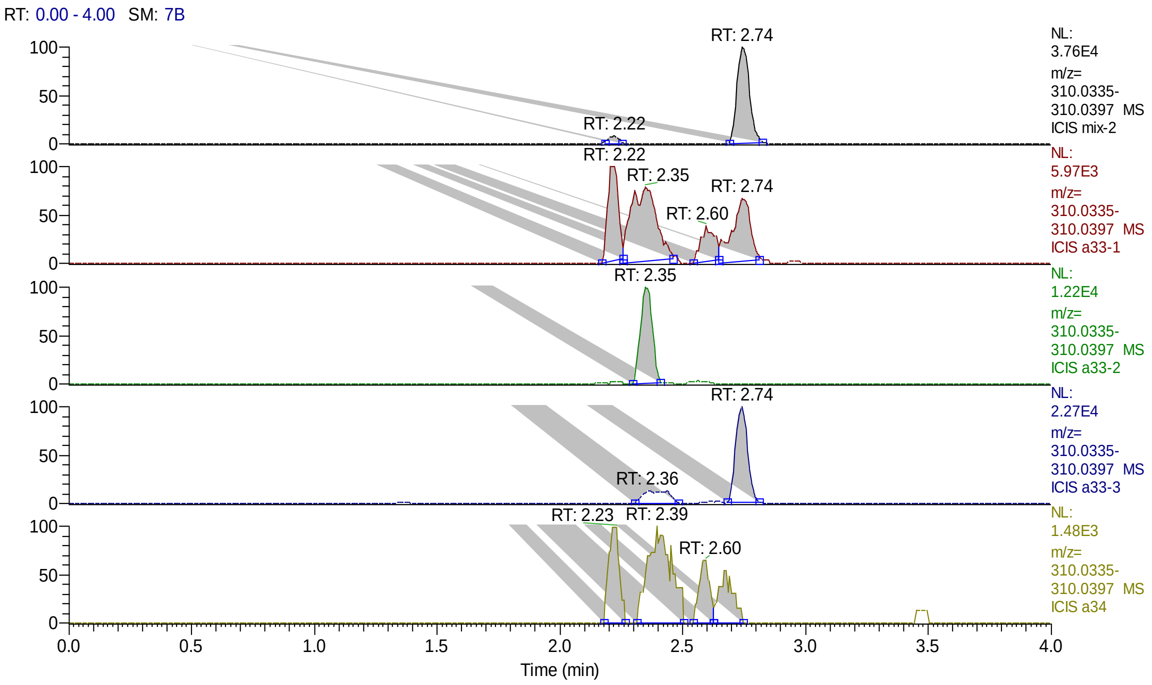


mix-2: standard AA II; a33-1: the fruits of *Ar. debilis*; a33-2: the herbs of *Ar. debilis*; a33-3: the roots and rhizomes of *Ar. debilis*; a34: *Ar. arborea*

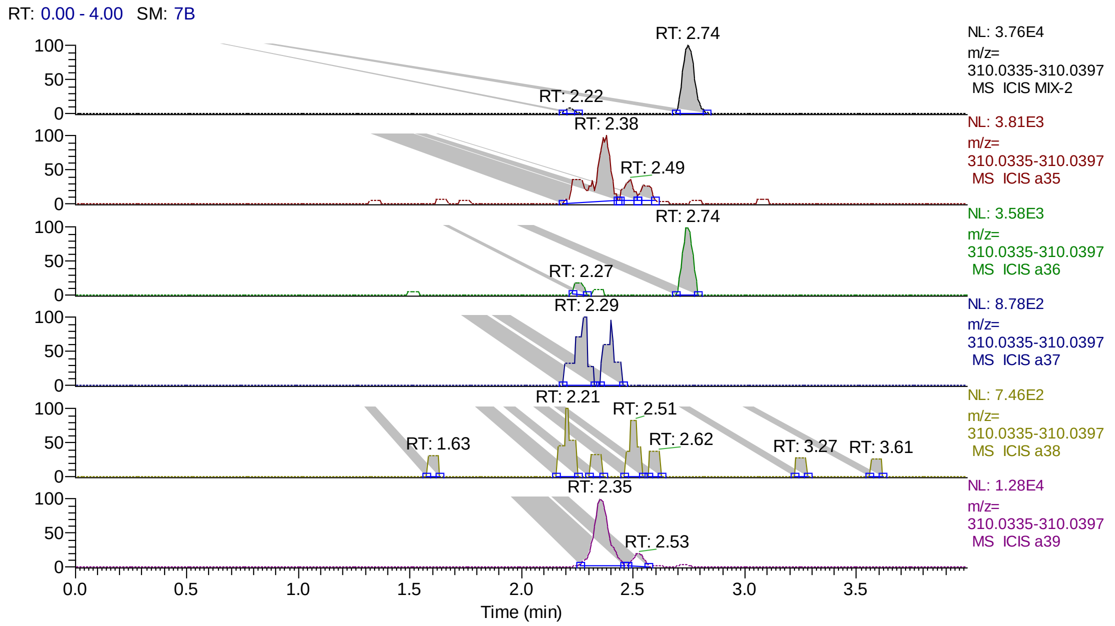


mix-2: standard AA II ; a35: *Ar. fimbriata*; a36: *Ar. gibertii*; a37: *Ar. gigantea*; a38: *Ar. grandiflora*; a39: *Ar. tagala*

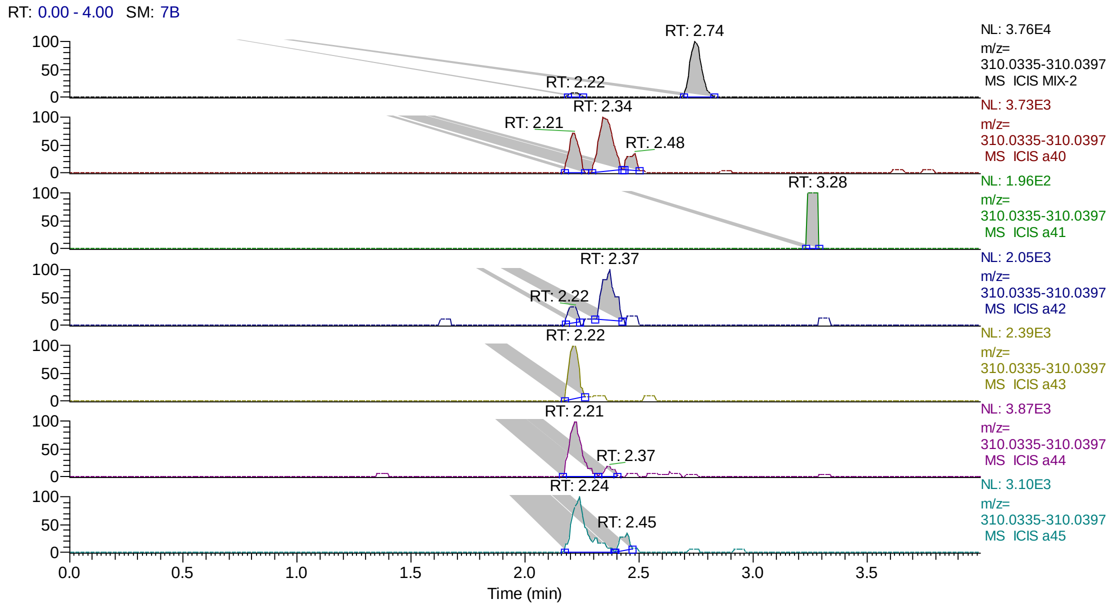


mix-2: standard AA II ; a40: *Ar. kwangsiensis*; a41: *Ar. elegans*; a42: *As. caudigerum*; a43: *As. caulescens*; a44: *As. campaniflorum*; a45: *As. pulchellum*;

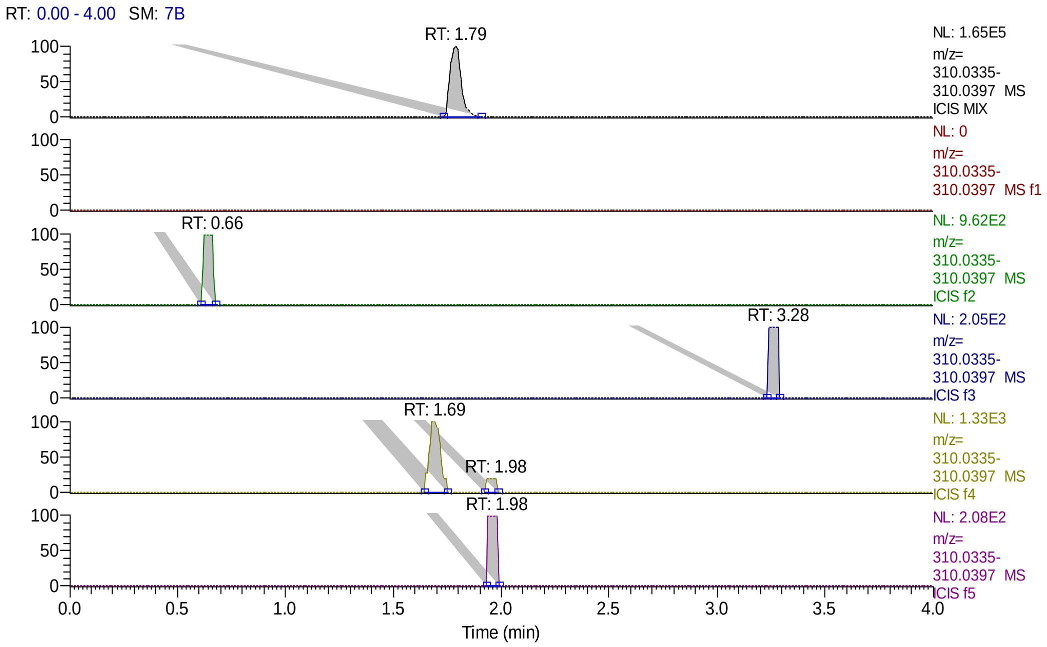


MIX: standard AA II ; f1: *Stephania tetrandra*; f2: *Cocculus orbiculatus*; f3: *Cocculus laurifolius*; f4: *Menispermum dauricum*; f5: *Akebia quinata* ;

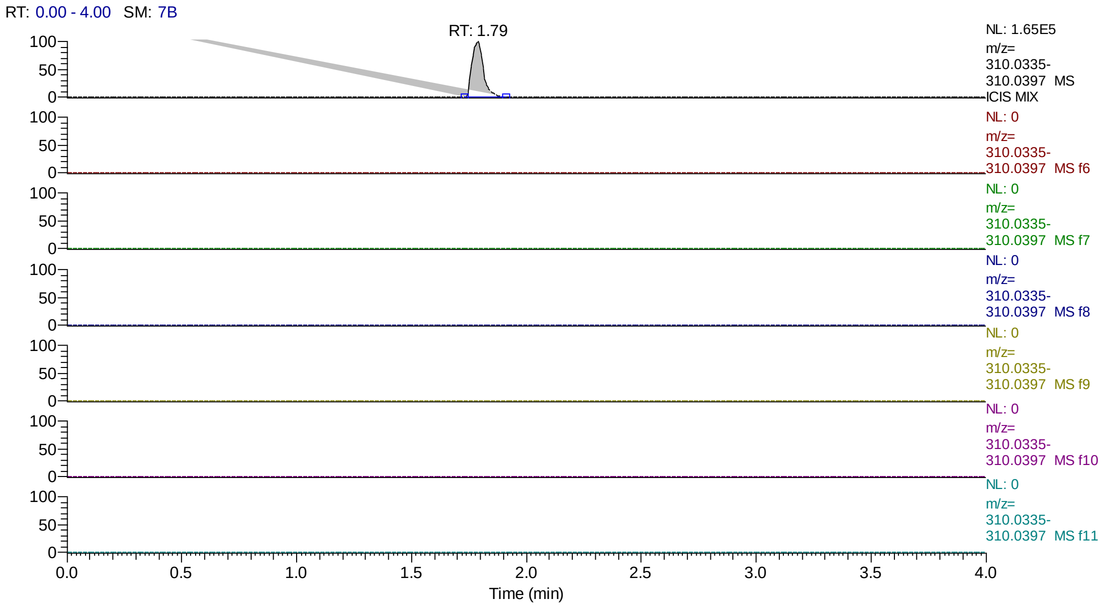


MIX: standard AA II ; f6: *Akebia trifoliata*; f7: *Clematis armandii*; f8: *Clematis argentilucida*; f9: *Clematis peterae*; f10: *Sabia yunnanensis*; f11: *Cardiocrinum cathayanum*;

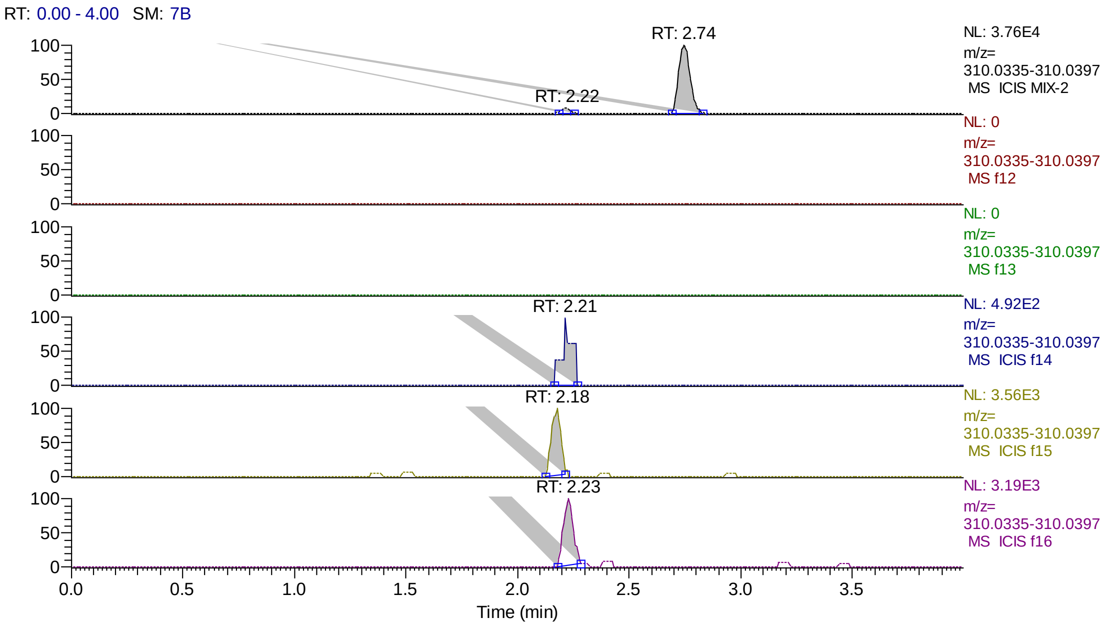


mix-2: standard AA Ⅱ; f12: *Diploclisia glaucescens*; f13: *Akebia trifoliata* var. *australis*; f14: *Clematis montana*; f15: *Sinomenium acutum*; f16: *Clematis chinensis*;

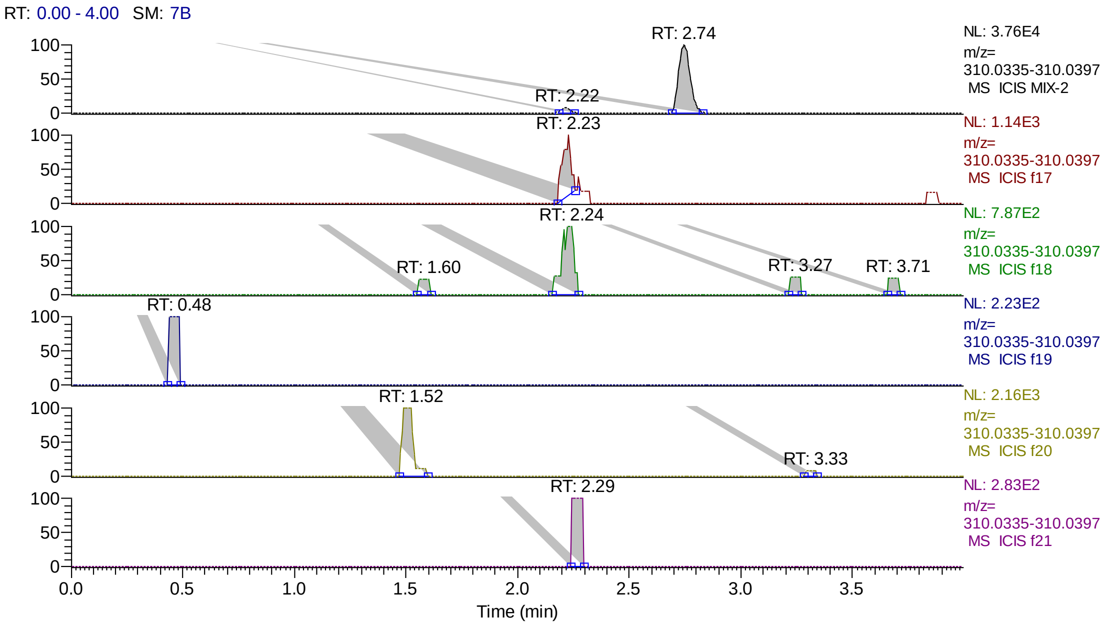


mix-2: standard AA Ⅱ; f17: *Clematis hexapetala*; f18: *Clematis manshurica*; f19: *Tetrapanax papyrifer*; f20: *Stachyurus chinensis*; f21: *Helwingia japonica*;

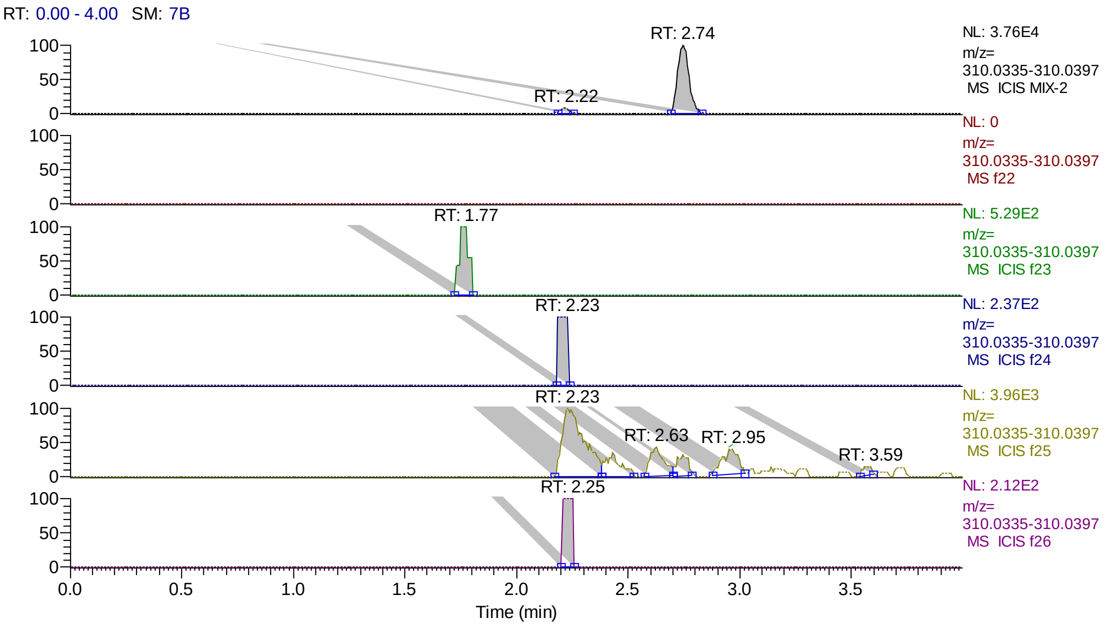


mix-2: standard AAⅡ; f22: *Aucklandia lappa*; f23: *Vladimiria souliei*;
f24: *Inula helenium*; f25: *Murraya paniculata*; f26: *Solanum lyratum*;

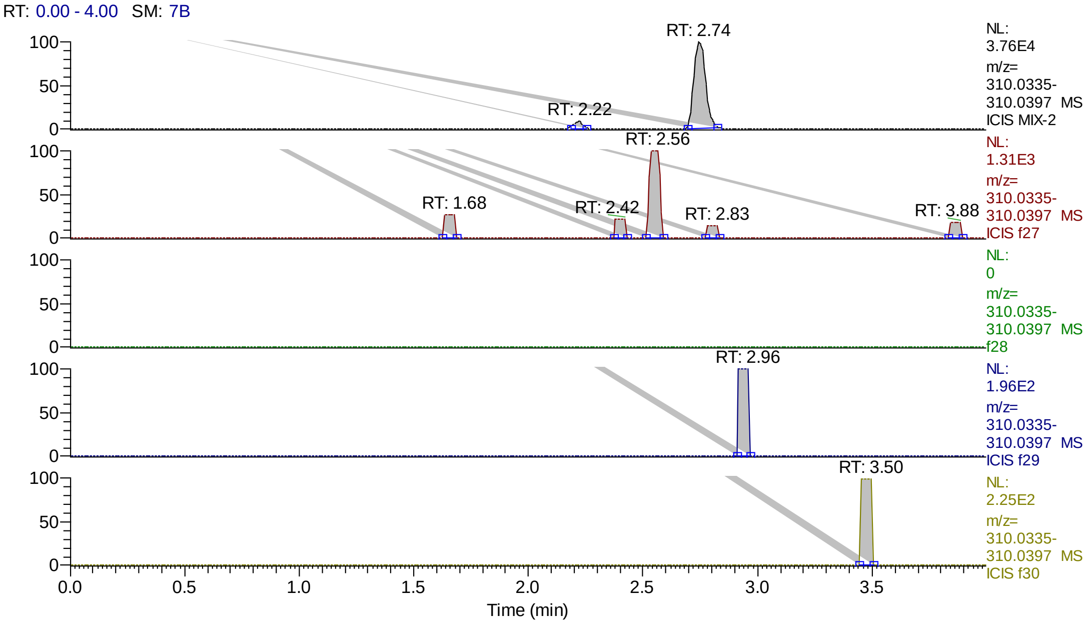


mix-2: standard AAⅡ; f27: *Pleione bulbocodioides*; f28: *Pleione yunnanensis*;
f29: *Cremastra appendiculata*; f30: *Tinospora sagittata*;

**Fig. S3. MS profiles of AAⅡ for all species in UHPLC-HR-MS analysis. AAⅡ was detected at *m/z* 310.0366.**
